# Assessment of Enzyme Inhibition: A Review with Examples from the Development of Monoamine Oxidase and Cholinesterase Inhibitory Drugs

**DOI:** 10.3390/molecules22071192

**Published:** 2017-07-15

**Authors:** Rona R. Ramsay, Keith F. Tipton

**Affiliations:** 1 Biomedical Sciences Research Complex, University of St Andrews, St Andrews KY16 8QP, UK; 2 School of Biochemistry and Immunology, Trinity College, Dublin 2, Ireland; ktipton@tcd.ie

**Keywords:** Alzheimer’s disease, *l*-deprenyl (selegiline), donepezil, galantamine, inhibitor constant, enzyme inhibition, multitarget-directed ligand (MTDL), rasagiline, rivastigmine

## Abstract

The actions of many drugs involve enzyme inhibition. This is exemplified by the inhibitors of monoamine oxidases (MAO) and the cholinsterases (ChE) that have been used for several pharmacological purposes. This review describes key principles and approaches for the reliable determination of enzyme activities and inhibition as well as some of the methods that are in current use for such studies with these two enzymes. Their applicability and potential pitfalls arising from their inappropriate use are discussed. Since inhibitor potency is frequently assessed in terms of the quantity necessary to give 50% inhibition (the IC_50_ value), the relationships between this and the mode of inhibition is also considered, in terms of the misleading information that it may provide. Incorporation of more than one functionality into the same molecule to give a multi-target-directed ligands (MTDLs) requires careful assessment to ensure that the specific target effects are not significantly altered and that the kinetic behavior remains as favourable with the MTDL as it does with the individual components. Such factors will be considered in terms of recently developed MTDLs that combine MAO and ChE inhibitory functions.

## 1. Introduction

With better healthcare across the world and an ageing population in the developed world, the demand and market for new treatments for complex biological malfunctions, such as neurodegeneration, dementia, and cancer, is enormous. Medicinal chemists engaged in modern drug discovery aim to design compounds that are able to interact with two or more targets to produce synergistic clinical effects from one compound, decreasing the risks and lack of compliance associated with combinations of several drugs.

Enzymes remain prime targets for drug design because altering enzyme activity has immediate and defined effects. Even with the increase in the use of drugs for receptors to modulate signals from outside the cell, 47% of all current drugs inhibit enzyme targets [1]. Recent multitarget-directed ligand (MTDL) approaches in medicinal chemistry have combined inhibitors of cholinesterases (ChE) and monoamine oxidases (MAO) to combat the loss of neurotransmitters in Alzheimer’s disease (AD), or of MAO with properties such as antioxidant and metal complexation for Parkinson’s disease (PD). For cancer treatment, compounds that combine kinase inhibitors or include cyclooxygenase inhibition to reduce inflammation [2] have been proposed. The MTDL inhibition of dihydrofolate reductase and thioredoxin reductase, enzymes belonging to two unrelated cellular pathways known to contribute to cancer cell growth [3], is another example.

Structure-based drug design for a single enzyme target has been facilitated, with crystal structures enabling the computational searches of huge chemical databases (reviewed in [4,5]) to identify lead compounds for refinement. With such large-scale computational approaches including analysis of off-target activity [6], combining suitable pharmacophores for enzyme combinations such as ChE, MAO and secretases (beta, gamma) for AD is entirely feasible. However, such compounds still have to be synthesised and tested experimentally, to confirm the predicted inhibitory effects against each of the targets, before progressing to the complex systems such as cell and in vivo models. The focus of this review is to highlight best practice in the assessment of enzyme inhibition for medicinal chemistry, drawing on examples from our own experience.

## 2. Inhibition of Neurotransmitter Breakdown in Neurodegenerative Disease

Signalling molecules in living systems are generated or released to have an effect mediated by a receptor and downstream pathways, but that signal must then be terminated. For signalling between neurons the time frame for signalling is short, so breakdown of the neurotransmitter is a key part of brain function. Acetylcholinesterase (AChE) terminates neuromuscular signalling efficiently. It is located on the post-synaptic membranes of neurons to hydrolyse acetylcholine released in inter-neuronal signalling. Cholinergic signalling, which is important in cognition, is lost as AD develops, so the first (and current) drugs used to combat the cognitive deficits of AD are acetylcholinesterase inhibitors (AChEIs). Butyrylcholinesterase (BChE) also hydrolyses acetylcholine, so it may be desirable that drugs against cognitive decline also inhibit that enzyme. Loss of other neurons is progressive in AD, decreasing dopamine, serotonin, and noradrenaline, all of which are metabolised by MAO, and or, by catechol-*O*-methyltransferase (COMT). MAO inhibitors have long been used as antidepressants, and an early trial against PD resulted in *l*-deprenyl (Selegiline) becoming accepted as a disease-delaying treatment for PD. Furthermore, improved dopamine release was shown to attenuate cognitive impairment in a triple-transgenic-mouse model of Alzheimer’s disease [7]. ChEs and MAOs are thus primary targets for most designs of MTDL for neurodegenerative diseases, resulting in many medicinal chemistry publications in the last 10 years (for example, [8,9,10,11,12,13,14]).

### 2.1. Monoamine Oxidases (MAO A and MAO B) as Drug Targets

There are two monoamine oxidase (E.C. 1.4.3.4) isoenzymes, MAO A and MAO B, which are encoded by separate genes on the X-chromosome. The MAO flavoproteins are located on the mitochondrial outer membrane, so metabolise amines inside the cell. The proportion of each MAO in the tissues varies, with the activities of the two forms being approximately equal in human liver. MAO A predominates in placenta and intestine and in dopaminergic and noradrenergic neurons, whereas MAO B predominates in platelets, glial cells, and serotonergic neurons [15,16,17]. Despite sharing the same catalytic centre structure, with substrate-orienting tyrosines leading to the covalently bound FAD [18], the two forms have different intrinsic activities (*k*_cat_ and *K*_m_ values) with any given substrate [19]. In the human brain, serotonin is mostly metabolised by MAO A, but dopamine is metabolised by both forms. In mice or humans lacking one form, all amines can be metabolised, to some degree, by the remaining form (reviewed in [20]).

Despite 70% sequence identity and overall structural similarity, the active sites have distinctly different shapes [21,22,23] and discriminate fine differences between substrates and inhibitors (reviewed in [18,24]). The kinetic constants (*k*_cat_ and *K*_m_) for the oxidation of the common neurotransmitters with oxygen at air-saturation (0.28 mM at 30 °C) are given in Table 1. The *K*_m_ for oxygen for cloned and purified human liver MAO A was determined to be 60 μM, but MAO A purified from human placenta gave a *K*_m_ of 6 μM [25] both assayed with kynuramine as the first substrate. It should be noted that the *K*_m_ value of MAO B for oxygen, determined at saturating amine concentrations, varies with the amine substrate due to the alternate pathways available during steady-state turnover [26,27].

The effect of the alternative pathways on kinetic behavior is most severe for MAO B. As well as giving different *K*_m_ values for oxygen with each substrate, the *K*_i_ value for an inhibitor also changes with the substrate used. However, detailed kinetic studies on MAO B can give not only the true *K*_i_ values but also additional information such as the binding of reversible inhibitors to both the oxidized and reduced forms of the enzyme [27]. The alternate pathways and the high *K*_m_ for oxygen in MAO B mean that the kinetic behavior can be complex for MAO B, as can the relationships between IC_50_ values and *K*_i_ (see later).

The major drugs for inhibition of MAO, originally developed as antidepressants are irreversible inhibitors. This means that new protein must be synthesised to replace the inactivated enzyme. The half-life of MAO B in the brain is 30–40 days [29,30], so the effect of these irreversible drugs is long lasting. For reversible inhibitory drugs, frequent dosing is required to maintain a high level of inhibition. Reversible drugs include the anti-depressant moclobemide (selective for MAO A) and safinamide (selective for MAO B), newly approved in Europe as Xadago for adjunct therapy for PD. Like many MAOI, safinamide has non-MAO B effects that enhance its clinical efficacy—it inhibits dopamine reuptake and also excessive glutamate release via sodium channel blockade [31]. The dissociation constant (*K*_d_) for safinamide binding to MAO B, measured by thermal-shift calorimetry, was 187.2 (±117.2) nM, in contrast to the “*K*_i_” value of 5 nM calculated from the IC_50_ (see below) in a coupled luminesence assay [32]. This difference emphasizes the distinction between the equilibrium binding to the oxidized enzyme (as measured by thermal calorimetry or calculated by molecular docking) and the kinetic IC_50_ that measures the effect of inhibitor binding on catalysis, which depends on the kinetic mechanism involved. This may include binding to the both the oxidized and reduced forms of MAO B, which can be quite different [33]. Ultimately, the *K*_i_ is the functional value that will apply in brain to decrease neurotransmitter breakdown.

Although much effort has been expended to design compounds that are selective for either MAO A or MAO B, the question of which enzyme should be inhibited is often ignored. MAO A is predominant in all neurons except serotonergic ones. After the neurotransmitter is taken back into the cell via the specific reuptake transporter (the normal mode of signal termination), MAO and the storage vesicle carrier (VMAT) compete for the neurotransmitter. When MAO A is inhibited in the neuron, more neurotransmitter will be returned to storage and be available for the next release and the rising cytoplasmic amine levels will impede uptake into the nerve terminals. Serotonin is a very poor substrate for MAO B, so the role of the presynaptic MAO B in serotonergic neurons may protect against exogenous amines. It has been shown, for example, that there is some non-specific uptake of neurotransmitters when monoamine levels are high. Thus, dopamine uptake into serotonergic neurons via the serotonin tranporter (SERT), results in complex interactions after MAO B inhibition [34]. However, the majority of MAO in the brain (80%) is present in the glial cells (including oligodendrocytes, astrocytes, ependymal cells, Schwann cells, microglia, and satellite cells). The MAO B in these cells metabolizes neurotransmitter amines that have diffused away from the synapse, amines that will be excreted either as the parent compound or, mainly, as the metabolites after the actions of MAO, catechol-*O*-methyltransferase (COMT) and the aldehyde dehydrogenases and reductases. Evidence from knock-out mice shows that loss of MAO A activity results in high serotonin and noradrenaline, and aggressive behavior [35]. Loss of MAO B results in very high concentrations of phenylethylamine but with no change in serotonin, noradrenaline or dopamine levels, and very little change in behavior apart from increased novelty-seeking [35].

The localization of MAO A and the effects of its knock-out or inhibition in animals are consistent with the observed antidepressant effects of MAO A inhibitors. Neuroimaging with harmine, a tight-binding MAO inhibitor, demonstrated increased MAO-A levels in the cortex, striatum and midbrain of people with major depressive disorder [36] that could decrease the overall levels of the monoamines in brain. In the clinic, the level of inhibition of MAO A that provides an effective antidepressant result is over 74% [37]. MAO A levels are increased by glucocorticoids providing a possible link to stress and depression [35,38,39]. Although MAO A inhibitors have proved to be effective as antidepressants, dietary amines, such as tyramine can cause a severe hypertensive response, often referred to as the ‘cheese reaction’, when intestinal MAO-A is inhibited [39]. Transdermal inhibitor formulations or, as discussed later, reversible inhibitors have been used to avoid this.

MAO A expression is vital for brain development and MAO A knock-out results in disruption of the serotonin levels that change during embryogenesis [40,41]. However, MAO A also modulates apoptosis during development [41] and is associated with induction of apoptosis in cells [42,43,44]. Links have been made between MAO A and neurodegeneration via the AD-associated protein presenilin-1 (PS-1). The PS-1 protein is part of the γ-secretase complex that produces amyloid-β (Aβ) peptides, which aggregate in AD. Protein-protein interaction between the endoplasmic reticulum protein PS-1 and MAO-A in mitochondria-associated membranes was demonstrated by co-immunoprecipitation, and low PS-1 was associated with increased MAO A in cells [45,46]. This direct protein link to AD suggests that MAO A should be considered as a target in AD, as well as in antidepressant drug design.

In ageing brain, MAO B increases as the proportion of glial cells increase relative to neurons. The consequent elevated levels of MAO B as well as other monoaminergic perturbations in Alzheimer’s brain [47] have suggested the value of incorporating MAO B inhibition into multitarget drugs to treat AD. MAO B inhibition has been combined with AChE inhibition, as described below, but there is, as yet, no drug in clinical use specifically to address monoaminergic systems in AD. In PD, the MAO B-selective inhibitors *l*-deprenyl and rasagiline are used in the clinic to delay the need for L-DOPA but also as adjunctive therapy to spare dopamine. In addition to generating dopamine for the remaining dopaminergic cells, L-DOPA is also accumulated into non-dopaminergic cells resulting in release of dopamine from sertonergic terminals and complex interaction between 5-HT and DA systems [48,49]. MAO B inhibition has also been incorporated with components to chelate iron and to reduce oxidative stress, giving the MTDL for PD, ladostigil [50]. Thus, for drugs to combat neurodegenerative disease, MAO B is currently an important monaminergic target.

### 2.2. Inhibitors of Monoamine Oxidases (MAO A and MAO B)

**Figure 1:**
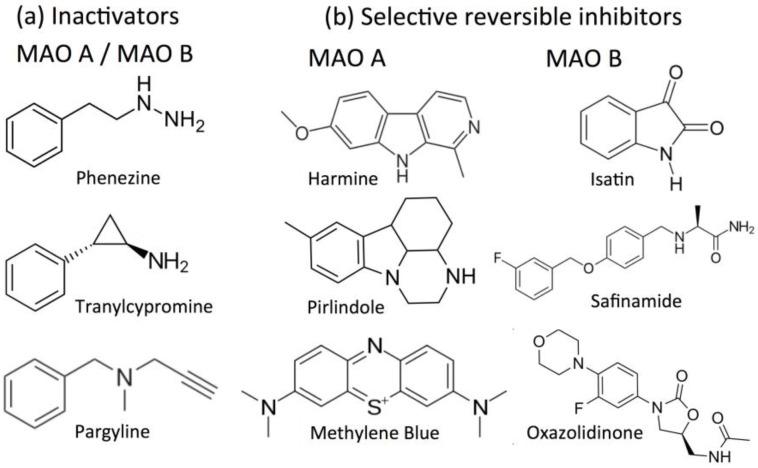
Structures of typical MAO inhibitors. (**a**) Irreversible inhibitor drugs; (**b**) Examples of reversible selective inhibitors.

Figure 1 shows examples of irreversible and reversible inhibitors of MAO.

#### 2.2.1. Irreversible Inhibitors

Three classes of compounds that inactivate MAO are represented in currently approved drugs.

##### Hydrazines

The first antidepressants were derived from the hydrazine drugs used to treat tuberculosis. Hydrazine derivatives, such as phenelzine (Figure 1a) are MAO substrates but the activated product can form a covalent bond to the N5 of the FAD cofactor [51]. Oxidation of the C-H bond of the hydrazine gives the hydrazone that is then hydrolysed to give the aldehyde [52]. Binda et al. [51] proposed that, for irreversible inhibition, the HN-NH bond is oxidized to give a diazene that can lose an electron to oxygen leaving a reactive radical that alkylates the N5 of the flavin. The process is not efficient, as 7 mol of oxygen are consumed (and so 7 mol of product liberated) per mol of enzyme inactivated.

##### Cyclopropylamines

Tranylcypromine (Parnate), which was introduced in the 1960s, inactivates MAO, with a slight preference for MAO B, but it inhibits MAO A in the gut sufficiently well in vivo to cause a hypertensive crisis unless dietary restrictions are followed [53]. Cyclopropylamines have been studied as inactivators of MAO [54,55,56] and of the epigentic-modifying flavoenzyme, enzyme LSD-1 [57]. Tranylcypromine (Figure 1a) also inhibits the copper-containing amine oxidases [58] and cytochrome P450 isoforms [59,60]. Although it has multiple effects in vivo, it is still in use for major depression [61].

##### Propargylamines

The first drug used in the propargyl class was the non-specific pargyline (Figure 1a). Subsequent development led to selective inactivators, clorgyline for MAO A and *l*-deprenyl and rasagiline for MAO B. *l*-Deprenyl and rasagiline are used for adjunct therapy of Parkinson’s disease. Both *l*-deprenyl and rasagiline inactivate MAO B at nanomolar concentrations, but can also inactivate MAO A at higher concentrations [30]. A polar anionic mechanism has been proposed from quantum-chemical calculations, where the flavin moiety acts as an electrophile and the bond is formed after deprotonation at the terminal carbon [62]. The acetylenic moiety of the propargyl group is particularly useful for incorporation into multi-target compounds because specificity is defined by the requirement for the catalytic activity of the MAO. Its further adaptation into multi-target compounds for neurodegenerative diseases has been reviewed [63].

#### 2.2.2. Reversible Inhibitors

The design of effective reversible inhibitors of MAO was driven by the dietary restrictions required during use of irreversible inhibitors of MAO A. Some reversible inhibitors of MAO are produced endogenously in the brain. The indole, isatin, inhibits MAO B [64], and the beta-carboline, harmine, is a potent inhibitor of MAO A (Figure 1b). The only current drug marketed for reversible inhibition of MAO A is moclobemide [65]. The anti-bacterial drug linezolid is a weak inhibitor [66], but many other oxazolidinones are potent reversible inhibitors of MAO [67,68], including the MAO-A selective inhibitor befloxatone [69]. The need to avoid inhibiting MAO A in the gut and the increase of MAO B in ageing brain has driven the search for potent reversible inhibitors of MAO B. Safinamide, an anticonvulsant now marketed for Parkinson’s disease, is an inhibitor of MAO B that is effective at nanomolar concentrations [70]. Many other new series of compounds have been designed and published in 115 medicinal chemistry articles in the last 5 years (Web of Science). A measure of the promise of some of these is summarized in a recent review of 27 patents between April 2012 and September 2014 covering 40 synthetic compounds in addition to natural products [71]. Taking one example, the coumarin scaffold has been thoroughly explored, producing numerous selective, reversible MAO B inhibitors of potential use in the development of multi-target agents [72].

### 2.3. Acetylcholinesterase (AChE) and Butyrylcholinesterase (BChE) as Drug Targets

Human tissues contain two enzymes that catalyse the hydrolysis of acetylcholine, acetylcholinesterase (AChE; EC 3.1.1.7) and butyrylcholinesterase (BChE; EC 3.1.1.8). The situation has been confused by the many other names that have been used for these enzymes. AChE is also known as true cholinesterase; choline esterase I; cholinesterase; acetylthiocholinesterase; acetylcholine hydrolase; acetyl-β-methylcholinesterase; AcCholE; whereas butyrylcholinesterase has also been called pseudocholinesterase; butyrylcholine esterase; non-specific cholinesterase; choline esterase II (unspecific); benzoylcholinesterase; choline esterase; butyrylcholinesterase; propionyl-cholinesterase; BtChEase. This can make it difficult to interpret some of the earlier literature on these activities. As the name cholinesterase has been used for both, it should be avoided except when used to cover both enzymes. Both enzymes are glycoproteins that can exist as membrane-bound or soluble forms.

AChE is a key enzyme in both central and peripheral cholinergic neurotransmission [73]. It is predominately membrane-bound and is found mainly at neuronal synapses in the central nervous system and at neuromuscular junctions, where it terminates the action of acetylcholine (Ach) that has been released, by nerve stimulation. The enzyme is also found associated with the erythrocyte membrane and in soluble forms. There is a single AChE gene in the human, but different mRNA splicing results in sequence variations at the C-terminal end of the enzyme that determine its membrane attachment. The commonest form expressed in mammalian systems is the ‘tailed’ variant in which there is a 40 residue C-terminal ‘T peptide’ that can associate with membranes either through the collagen type anchoring protein, collagen Q (ColQ), or through the transmembrane protein, PRiMA (proline-rich membrane anchor) [74]. The PRiMA-associated form is the major AChE present in brain [75], whereas ColQ attachment is found at neuromuscular junctions. In contrast, the attachment to the erythrocyte membrane is through a glycophosphatidylinositol (GPI) anchor [76]. Other variants, without the T-peptide result in soluble forms of the enzyme, which like the membrane-bound forms may be monomeric dimeric, or tetrameric. All these forms appear to possess the same catalytic domain.

**Figure 2:**
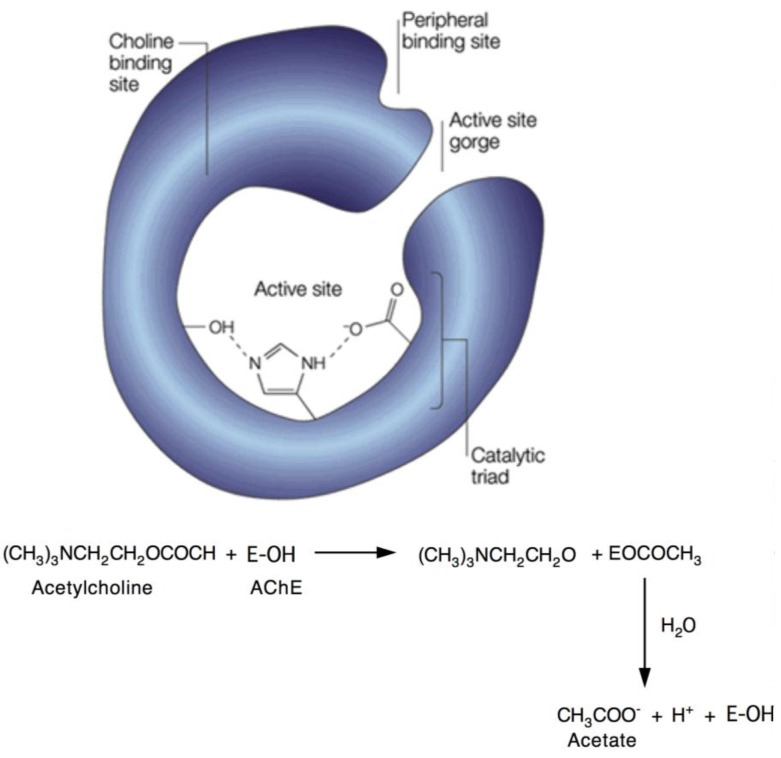
Acetylcholinesterase model illustrating the active-site gorge with the catalytic triad of His-Ser-Glu located in a hydrophobic environment. The peripheral anionic site (PAS) is shown close to the entrance to the active-site gorge, reproduced with permission from Soreq and Seidman [99]. The lower part shows the pathway of acetylcholine hydrolysis. For simplicity, the initial non-covalent enzyme-substrate complex has been omitted.

AChE catalyses the hydrolysis of Ach with high efficiency, but the location of its catalytic site in a deep cavity, or gorge (Figure 2), results in a lesser ability to cleave esters with bulkier acyl groups [77]. In contrast to AChE, butyrylcholinesterase, which has a wider gorge, is a less specific esterase which can cleave various esters with bulkier acyl groups [78,79]. Table 2 summarizes some of the known specificity differences between these two enzymes. BChE also has aryl acylamidase activity that catalyses the hydrolysis of acyl amides of aromatic amines. The synthetic compound σ-nitroacetanilide is often used to determine this activity [80], which seems to involve the same catalytic site as that involved in choline ester hydrolysis. A physiological substrate for this activity has not yet been identified.

BChE is widely distributed in human tissues. In the human brain, it is expressed in some populations of neurons that are distinct from those associated with AChE [81], and it is also present in glial cells. Serum levels of BChE are much higher than those of AChE. Like AChE, BChE exists in soluble and membrane-bound forms. The soluble forms comprise monomers, disulfide-linked dimers and tetramers (the G1, G2 and G4 forms). The membrane bound forms are linked via PRiMA or ColQ) [82]. The physiological functions of BChE are still subject to some speculation and much earlier work was devoted to its inhibition to allow Ach to be determined. Tetraisopropyl pyrophosphoramide (iso-OMPA) and ethopropazine (profenaminerofenamine) have been used for this purpose [81]. Its activity towards succinylcholine (suxamethonium), which has been used as a muscle relaxant during surgery, resulted in the identification of individuals with an unusual variant of BChE that had reduced activity and does not metabolize succinylcholine efficiently [83], leading to a prolonged and potentially fatal muscle-paralyzing action of this drug.

The fact that such individuals appeared to exhibit no other deleterious effects, strengthened the view that the main role of role of BChE was that of a scavenger in peripheral tissues. In the brain it has been suggested that BChE cooperates with AChE in regulating ACh neurotransmission if the ACh levels rise too high, particularly since AChE is inhibited by high concentrations of its substrate [84]. Although BChE activity is lower than that of AChE in the normal human brain, the BChE/AChE ratio is greatly increased in AD [85,86], suggesting that inhibition of BChE may become important as AD progresses. This has raised the hypothesis that inhibitory action on both ChEs could lead to improved therapeutic benefits [87] or that inhibition of BChE alone might be beneficial [88].

In evaluating possible effects of inhibiting these enzymes, it must be considered that they have also roles that are not strictly concerned with neurotransmission. Acetylcholine has been shown to be involved in suppression of cytokine release through a “cholinergic anti-inflammatory pathway” suggesting a role in the role the immune system [89]. However, it appears that AChE itself may also have a non-enzymic role in modulating stress responses [90]. Alternative splicing results in the stress-induced monomeric and soluble AChE-R variant in which the C-terminal region does not fold. This variant and the C-terminal 26 amino-acid peptide (ARP), which can be cleaved from it and can penetrate cells, stimulates cell proliferation.

Both AChE and BChE have been found associated with amyloid plaques and AChE appears to promote amyloid Aβ fibril formation, whereas the AChE-R variant appears to attenuate it [91]. The involvement of AChE in this process appears to involve a binding site situated at the rim of the active site gorge that is known as the peripheral anionic site (PAS). This appears to bind and orient acetylcholine facilitating its entry to the active site gorge. This interaction with the PAS also facilitates catalysis and is responsible for the inhibition that occurs at high substrate concentrations [92,93]. Amyloid-β protein binding to the PAS has been shown to accelerate aggregation, and, furthermore, compounds that bind to the PAS inhibit this process [94,95]. AChE also appears to play a role as an adhesion protein in aspects of nervous system development in a process that involves the PAS (see [95]). Clearly this may have consequences for neonatal exposure to some AChE inhibitors. An additional AChE variant, formed through an alternative promotor site, which has an *N*-terminal extension, has been shown to promote apoptosis [96].

The situation concerning BChE appears less complicated but many mutations have been reported. The, relatively common, variant termed BChE-K, in which there is an alanine-to-threonine substitution in the C-terminal region, has been of particular interest. It has reduced BChE activity and, whereas BChE has been reported to inhibit amyloid fibril formation, this ability is weakened in the BChE-K variant [97]. The literature on whether or not this constitutes a risk factor in AD or its progression has been contradictory [98]. BChE also has a peripheral site, which is somewhat smaller than that of AChE [79].

### 2.4. Inhibitors of Acetylcholinesterase (AChE) and Butyrylcholinesterase (BChE)

The observations that presynaptic ACh levels and the activity of its synthetic enzyme, choline acetyltransferase are decreased in AD resulted in the use of AChE inhibitors to treat the cognitive dysfunction. AChE inhibitors have also been used in the treatment of myasthenia gravis and as a CNS stimulant (analeptic) following the early observation that tacrine (1,2,3,4-tetrahydro-9-acridinamine) caused rapid arousal in experimental animals after morphine administration [100]. Tacrine was subsequently shown to be a reversible inhibitor of both AChE and BChe (see [101,102]). This was the first drug approved in the USA for therapy in Alzheimer’s disease. Despite moderate success, its use was subsequently discontinued because of adverse effects. Along with several other cholinesterase inhibitors, it has also been marketed as a cognitive enhancing (nootropic) agent.

Inhibitors of AChE may interact with the active site, the PAS or both. Tacrine appears to bind to both sites in AChE, resulting in mixed-type inhibition, whereas it is a competitive inhibitor of BChE, interacting solely at the active site region [101]. Active-site directed irreversible inhibitors involve mechanisms based on the normal catalytic mechanism of the cholinesterases. This involves a serine residue at the active site, which interacts with adjacent histidine and glutamate residues that increase its nucleophilicity (the catalytic triad). As shown in Figure 2, this splits acetylcholine to form an acetyl enzyme plus choline. The acetyl enzyme is subsequently hydrolysed to regenerate the native enzyme plus acetate.

**Figure 3:**
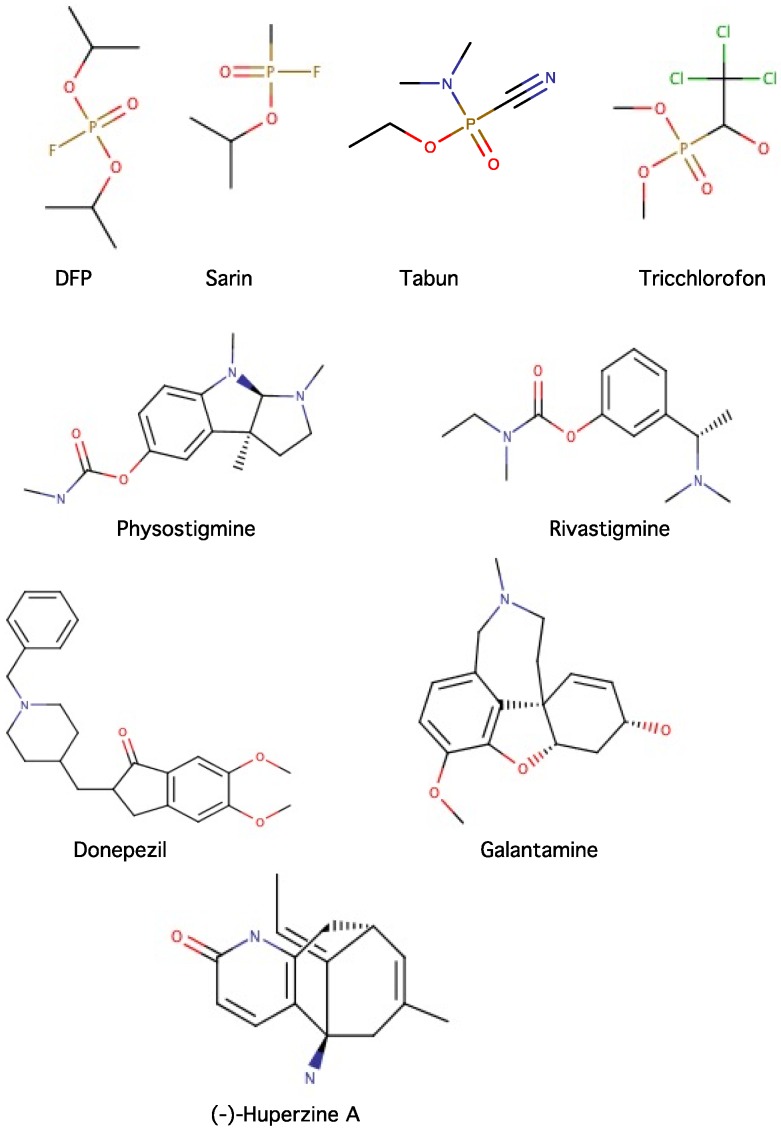
Some acetylcholinesterase inhibitors discussed in the text. The structures were produced using MarvinSketch from ChemAxon (http://www.chemaxon.com).

The organophosphate (OP) ‘nerve gases’, such as DFP, Sarin, Soman, Tabum and VX, which were originally developed as chemical warfare agents, behave as ‘suicide substrates’, operating according to the pathway shown in Figure 3. The phosphorylated intermediate may be hydrolysed, to regenerate the free enzyme, or dealkylated to give an irreversibly inhibited species [103,104]. This dealkylation process is often termed ‘ageing’. The relative rates of these two processes depends on the nature of the organophosphate. Total inhibition of the AChE by such compounds can be fatal, as the loss control of respiratory muscles results in asphyxiation. Survivors of nerve agent poisoning often suffer chronic neurological damage [105]. This is termed organophosphate-induced delayed neuropathy and appears to result from the inhibition of an enzyme that was named neuropathy-target esterase (NTE). NTE was found to catalyse the hydrolysis of phenylvalerate in vitro. Although the activity towards phenylvalerate has been used to assay NTE, that is not a specific assay since BCHE is also active towards that substrate [106]. It is now recognized that NTE is a member of the phospholipases A2 (PLA2) family enzymes that do not require Ca^2+^ for activity (the Ca^2+^-independent PLA2s or iPLA2s). The iPLA2s or patatin-like phospholipases (PNPLAs) are intracellular enzymes that, and contain lipase (GXSXG) and nucleotide-binding (GXGXXG) consensus sequences. The mechanisms involved remain somewhat unclear but it appears that an ‘ageing’ process may be involved in their inhibition, and that the effects may be owing to a change of iPLA2 function rather that simple inhibition of the esterase activity [107,108].

Despite the toxicity, the organophosphate DFP and has been used in the therapy of glaucoma and myasthenia gravis [109] and DFP and trichlorfon (metrifonate) has been used in AD therapy [110] and in the treatment of schistosomiasis [111].

**Scheme 1:**
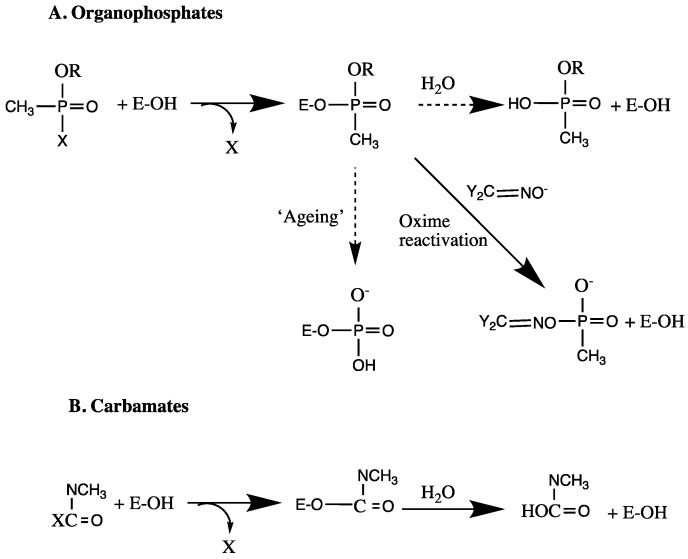
Pathways involved in the interaction of acetylcholinesterase with (**A**) organophosphates and (**B**) carbamates. The initial noncovalent enzyme-organophosphate and enzyme-carbamate complexes have been omitted.

The dangers of OP insecticides and herbicides led to them being replaced by carbamates (carbamic acid derivatives). These react in a similar way to OPs, forming a carbamylated serine intermediate that is then hydrolysed to regenerate the free enzyme, as shown in Scheme 1 [103]. The rate of this decarbamylation process depends on the nature of the carbamate [112,113], but complete recovery usually occurs in less than an hour and, unlike the OPs there is no ‘ageing’ process leading to irreversible inhibition. For this reason, the carbamates are often regarded a pseudo-irrreversible (or pseudo-reversible) inhibitors. Many behave similarly towards BChE, but some, such as the substituted phenothiazine carbamate derivatives are simple reversible inhibitors of that enzyme [112]. They do not induce delayed neuropathy, and have been shown to protect against neuropathy induced by OPs when administered before-hand, but exacerbate it if administered after. Pyridostigmine which is decarbamylated rather rapidly has been used for this purpose to protect against OP poisoning [114].

Carbamates have a long history. The naturally occurring carbamate physostigmine was first isolated from the calabar bean (the seed of the plant *Physostigma venenosum*) in 1864, and its structure was known by 1923 (see [115]). It has been used to treat glaucoma and myasthenia gravis, and was thought to have some value in the treatment of AD (see [116,117]). However, concerns about its toxicity as well as its short half-life in vivo restricted its use and stimulated the development of potentially safer and more effective analogues [117]. Of these, eptastigmine [118] has shown some promise, but rivastigmine (Exelon) (Figure 3) which inhibits both AChE and BChE has received approval in several countries for the treatment of mild-to-moderate AD [119]. Other inhibitors include:

Donepezil (Aricept) is a piperidine-derivative (Figure 3) [120] , which has also gained approval for use with AD. It is a reversible AChE inhibitor that binds to the peripheral anionic site, and does not affect BChE. It has symptomatic effects in AD treatment but, like rivastigmine, it also appears to delay the deposition of amyloid plaque (see [94]) or to enhance plaque removal [121]. It has a relatively long half-life in vivo (about 70 h), reducing the need for too frequent dosage.

Galantamine (Razadyne; Nivalin) is an alkaloid (Figure 3) that was isolated from the plant Galanthus woronowii, which can be used for the treatment of mild to moderate AD. It is a reversible competitive inhibitor of AChE that interacts with the PAS, as well as with the aromatic gorge [122]. It is about 5 times less potent as an inhibitor of BChE [112]. Galantamine also affects nicotinic cholinergic receptors increasing their sensitivity to Ach [123], which may be an added benefit in AD.

Huperzine A (Figure 3) is an alkaloid that was first isolated from the club moss Huperzia serrata, which has been a traditional ‘folk-medicine’ remedy in in China. It binds to the PAS, but may also penetrate the active site gorge [124]. It is much less potent as an inhibitor of BChE. It appears to have several additional targets, in protecting mitochondrial function, reducing the tissue labile iron pool and behaving as an antioxidant. [125]. Although Huperzine A is licenced for use as a drug for AD and vascular dementia in China, it is only available as a nutraceutical in many other countries. This may have hampered detailed international clinical trials and account for the conflicting evidence that is presently available on its effectiveness [126,127].

There have been many, sometimes conflicting reports on the comparative efficacy of donepezil, galantamine, and rivastigmine. A Cochrane Systematic Review concluded that there was little to choose between them in terms of their value in the therapy of mild-to-moderate AD and that the choice might be dictated by the side-effects experienced by an individual [128].

### 2.5. Design of Compounds that Inhibit Both MAO and ChE

**Figure 4:**
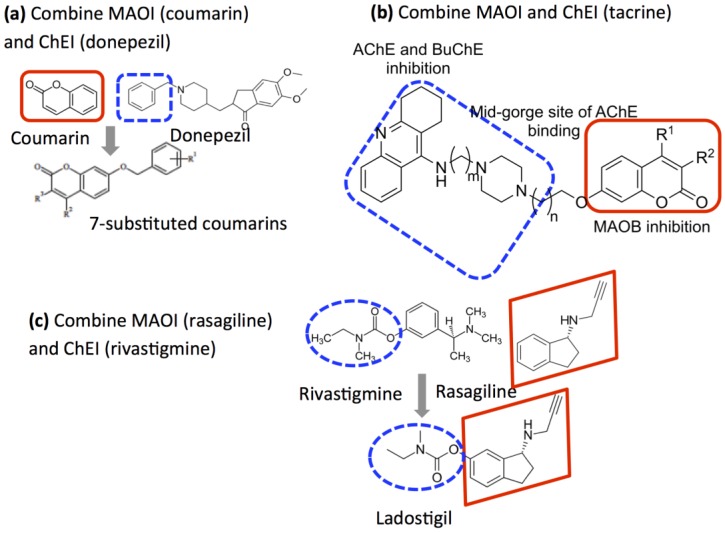
Combining existing drugs to produce multi-target compounds. (**a**) The core structure of donepezil, an anticholinesterase drug used for AD cognitive effects, is combined with coumarins that inhibit MAO [14]; (**b**) the discontinued AD drug, tacrine, combined with coumarin [129]; (**c**) combining the propargyl MAOI rasagiline with the carbamate moeity of the AD drug rivastigmine [130].

**Figure 5:**
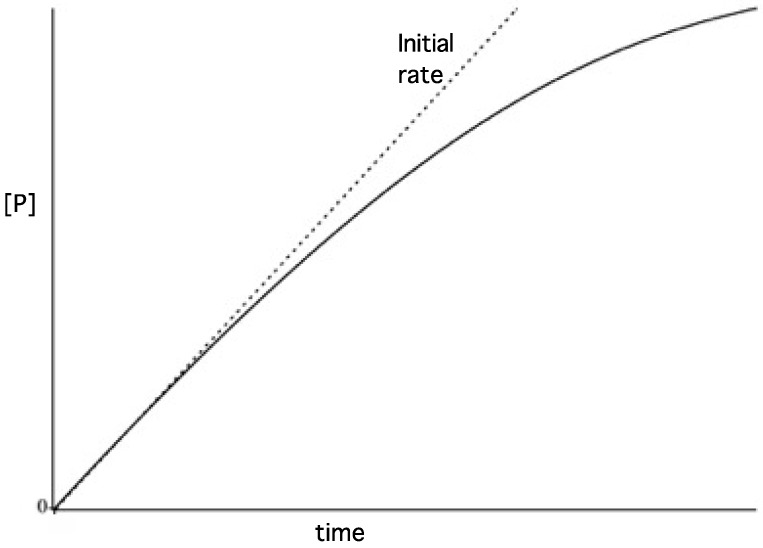
Time-course of an enzyme catalysed reaction, measuring the concentration of product.

The starting point for rational design of dual inhibitors of MAO and ChE has been existing drugs and known pharmacophores. Figure 4 shows the strategy followed in some recent publications to combine anti AD drugs donepezil or tacrine that inhibit ChEs with coumarin structures that inhibit MAO. The combination of donepezil and coumarin shown in Figure 5 met with some success with the best compound showing micromolar AChE and BChE inhibition (IC_50_ = 9.10 μM and 5.90 μM, respectively), and selective hMAO-B inhibition (IC_50_ = 0.30 μM), but the potency on the four enzymes varied widely across the series [14]. Using tacrine as in the example in Figure 4b was less successful because the large size decreased the inhibition of MAO (IC_50_ = 10–100 μM), although the inhibtion of ChEs remained in the sub-micromolar range [129].

## 3. Measurement of Enzyme Activity

Many books and articles have been written about enzyme behavior and assays. A recent online book is a useful and convenient resource for guidance on the evaluation of enzyme inhibition [131]. Here, we outline the general principles for obtaining reliable information from enzyme assays, then consider the specific assays and complications for MAOs and ChEs.

### 3.1. Assay Procedures

The activity of an enzyme may be measured by determining the rate of product formation, or substrate depletion, during the reaction. In cases where it is necessary to use high substrate concentrations, it is often more sensitive to measure product appearance, rather than substrate disappearance, because of possible inaccuracies in determining a small decrease from a large initial value. As discussed below for the cholinesterases and monoamine oxidases, there may be several alternative assay procedures available and the choice between them may be made on the grounds of convenience, throughput, cost, the availability of appropriate equipment and reagents, and the level of sensitivity required. The general principles and behavior of enzyme assay procedures have been reviewed elsewhere [132,133,134] and the recipes for many specific MAO [135] and AChE [136] assays have also been presented. There are two main types of assay procedure:

- Continuous assays that monitor changes in reactant concentrations in real time.
  (a) Direct, in which the decrease in substrate or increase in product is measured, e.g., the spectrophotometric determination of benzaldehyde production from benzylamine, or the use of the oxygen electrode in MAO assays.
  (b) Indirect, in which additional reactions are used to convert a product into something that can be easily monitored. The Ellman assay for AChE and peroxidase-coupled assays for MAO and AChE are examples.
- Discontinuous (sampling) assays. These involve stopping the reaction after fixed time(s) before separating the product for quantification. Radiochemical assays and those based on HPLC for AChE and MAO fall into this class.

Each type has advantages and disadvantages and the choice may depend on the intended use. Direct, continuous assays are useful for kinetic studies. Discontinuous assays are often favoured for high-throughput screening, and sometimes direct assays are used discontinuously for that purpose.

### 3.2. Reaction Progress Curves

The time-course of product formation, or substrate depletion, is often curved, as shown in Figure 5. It is initially linear but the rate declines at longer times. This fall-off could result from one or more causes (see [132,133,134] for more detailed discussion), including:

1. Substrate depletion: The reaction may be slowing down because the substrate is being used up. As the substrate concentration falls the enzyme will become less and less saturated and the velocity will fall, tending to zero when all the substrate is used.
2. Approaching equilibrium: A reversible reaction may be slowing down because it is approaching equilibrium, where the rate of the backward reaction (converting product to substrate) will increase until, at equilibrium, it is equal to the rate of the forward (substrate to product) reaction and no net rate will be observed.
3. Product inhibition: Products of enzyme-catalysed reactions are frequently reversible inhibitors and their accumulation can result in a decreasing reaction rate.
4. Instability: One of the components of the assay system may be unstable, losing activity or breaking down during the assay. This may be the enzyme itself or one of the substrates.
5. Time-dependent inhibition: Some enzyme substrates are also time-dependent irreversible inhibitors, sometimes referred to as ‘suicide-substrates’ (see Section 3.8 for discussion).
6. Assay method artifacts: If the specific detection procedure used ceases to respond linearly to increasing product concentrations, this can lead to a decline in the measured rate of the reaction with time (see [132]).
7. Change in assay conditions: If the assay conditions are not constant the rate of product formation might be expected to change. If, for example, the reaction involves the formation or consumption of hydrogen ions, the pH of the reaction mixture may change during the reaction, unless it is adequately buffered. If this resulted in a change of pH away from the optimum pH of the reaction this could lead to a decrease in the rate of the reaction.

Coupled assays, which continuously remove one of the products, should prevent any decline from cause 2 and, possibly, 3 from occurring.

At very short assay times none of these effects should be significant. Thus, if the initial, linear, rate of the reaction is determined (see Figure 5), these possible complexities can be avoided. The linear portion of an assay is often long enough to allow the initial rate (or the initial velocity; *v*) to be estimated by drawing a tangent to (or taking the first derivative of) the early part of the progress curve. It has sometimes been assumed that restricting measurements of reaction rates to a period in which less than 10–20% of the total substrate consumption has occurred will give an accurate measure of the initial rate. However, the variety of possible causes for non-linearity indicates that such an assumption may not be valid.

In cases where curvature makes it difficult to estimate the initial rate accurately, it may be possible to do so by fitting the observed time-dependence of product formation to a polynomial equation and deriving the initial slope at *t* = 0. Graphs of product concentration/time against either time or product concentration will intersect the vertical axis at a point corresponding to the initial rate (see [134]).

**Figure 6:**
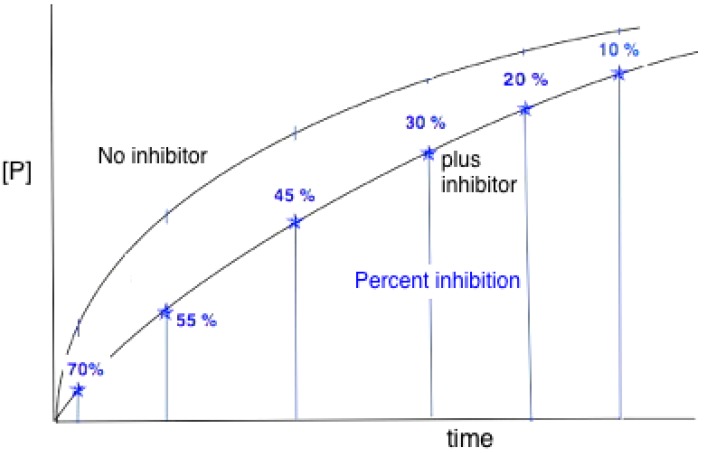
Time-course of an enzyme catalysed reaction in the presence of a competitive inhibitor. Values are shown for the percentage inhibition that would be calculated if the extent of reaction were measured at different times in the absence and presence of inhibitor.

The problems associated with failure to measure initial rates can be illustrated by the example shown in Figure 6. This exemplifies the fact that detailed controls are always necessary with discontinuous or batch assays, which involve determining the extent of the reaction after a fixed time, are to be used. It is essential to ensure that the time chosen gives a true measure of the initial rate under all conditions that are to be used.

### 3.3. Initial Rates and Coupled Assays

**Figure 7:**
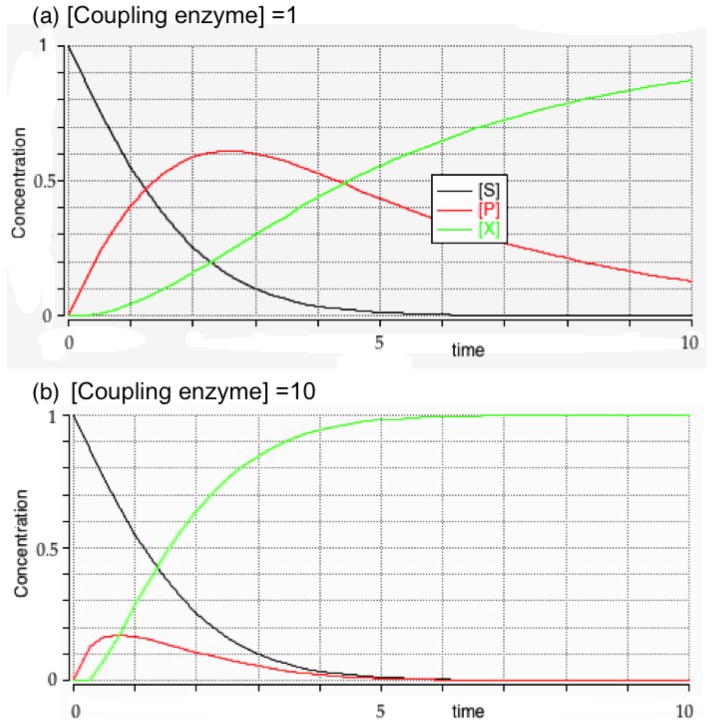
Behavior of a coupled-enzyme assay. In the example shown, the true rate of the reaction catalysed by the enzyme under study, measured by the formation of the end-product (X in green) will only be measured when its product (P in red) concentration is constant owing to a balance between it rate of formation and its removal by the coupling reaction. The activity of the coupling enzyme in the bottom panel (**b**) is 10 times greater than that in the top panel (**a**), giving a detection rate close to that of product formation. (Data simulated with the program Berkeley Madonna; http://www.berkeleymadonna.com/.)

A coupled assay can be represented by the simple equation:

(1) $$\mathrm{Substrate}\;\overset{\;\mathrm{Enzyme}\;1}{\to }\;\mathrm{Product}\;\overset{\;\mathrm{Enzyme}\;2}{\to }X$$

where the formation of the final product X is used to determine the activity of Enzyme 1. If assays of this type are to yield valid results it is essential that the coupling enzyme(s) used never becomes rate-limiting so that the measured rate is always that of the enzyme under study. The velocity of the reaction catalysed by a coupling enzyme will depend upon the substrate concentration available to it. Since this is produced by the activity of the enzyme under study, there will be very little of it available during the early part of the reaction and, thus, the coupling enzyme will be functioning at only a small fraction of its maximum velocity. As the reaction proceeds the concentration of the intermediate substrate will increase which will, in turn, allow the coupling enzyme to work faster. Thus, the rate of the coupling reaction will increase with time until it equals the rate of the reaction catalyzed by the first enzyme. At this stage, the concentration of the intermediate product will remain constant because of a balance between the rate of its formation, by the enzyme under study, and the rate of its removal by the coupling enzyme. This behavior of coupled enzyme assays is illustrated in Figure 7. There will be a lag in the rate of formation of the product of the coupled reaction. It is, of course, necessary to minimize this lag period, which is often referred to as the coupling time, because of the possibility that the reaction catalysed by the first enzyme will have started to slow down before the coupling enzyme has reached its steady-state velocity (Figure 7a). In such a case, the coupled assay would never give an accurate measure of the activity of the enzyme under study.

The efficiency with which a coupling enzyme can function will depend on its *K*_m_ value for the substrate being formed. The lower its *K*_m_ the more efficiently it will be able to work at low substrate concentrations. The lag period can also be reduced by increasing the amount of the coupling enzyme used, so that it can catalyse the reaction more rapidly at low substrate concentrations. The higher the *K*_m_ of the coupling enzyme the greater the amount of it will be required to produce the same lag period. Thus, it is important to characterize the performance of a coupled assay to ensure that it gives an accurate measure of the activity of the enzyme under study.

Usually this can be done experimentally by checking that the measured velocity is not increased by increasing the amount of the coupling enzyme present and is proportional to the amount of the first enzyme present, at all substrate concentrations, and under all conditions that are to be used. Generally, this is achieved by having a very large excess of the coupling enzyme(s) present. It is possible to calculate the amount of a coupling enzyme that must be added to give any given coupling time see [137] and such calculations may be useful in saving the expense of adding too much of the coupling enzyme(s). It must, however, be remembered that it will be necessary to re-check that a coupled assay is performing correctly each time the assay conditions are altered, since these may affect the behavior of the coupling enzyme(s). The purity of the coupling enzyme(s) and substrates used should also be checked. Since these are used in relatively high concentrations, even a small degree of contamination with other enzymes, or substrates that might affect the reaction under study could become important.

The Ellman assay for cholinesterase can be regarded as a coupled assay although the thiol produced is detected by a chemical reaction:

Thiol + DTNB → Disulfide + TNB(2)

The rate of TNB formation (R) will be given by the second-order rate constant *R* = *k* [Thiol] [DTNB] and since the DTNB concentration is much higher than that of the thiol formed this simplifies to the *pseudo* first-order rate of *R* = *k*’ [Thiol], where the apparent first-order rate constant is *k*’ = *k* [DTNB]. At the steady-state when the rate of thiol production is balanced by its removal, the concentration of thiol will be constant and hence the detection system will be governed by the zero-order equation *R*’ = *k*’_app_. Thus, under these conditions the rate of TNB formation will correspond to the rate of substrate hydrolysis. The rate constant for the thiol/DTNB is sufficiently large to ensure this under most conditions [138].

### 3.4. Expression of Enzyme Activity

Since enzymes are catalysts they are normally present at very much lower concentrations than their substrate(s). Therefore, the initial velocity of the reaction would be expected to be proportional to the concentration of the enzyme. Thus, activity of an enzyme can be expressed quantitatively by the ratio (velocity/enzyme concentration). This facilitates comparison of data obtained with the same enzyme from different laboratories, assessment of the effects of physiological or pharmacological challenges to cells or tissues, monitoring the extent of enzyme purification and comparison of the activities of different enzymes, or of the same enzyme from different sources or with different substrates. The most commonly used quantity is the Unit, sometimes referred to as the International Unit (IU) or Enzyme Unit. One Unit of enzyme activity is defined as that catalysing the conversion of 1 μmol substrate (or producing 1 μmol product) in 1 min. The specific activity of an enzyme preparation is the number of Units per mg protein. Since the term “unit” has also sometimes been used to refer to more arbitrary measurements of enzyme activity, it is essential that it is defined in any publication. If the molecular weight of an enzyme is known, the molecular activity, defined as the number of Units per μmol of enzyme; i.e., the number of mol of product formed, or substrate used, per mol enzyme per min, is sometimes used. This may not correspond to the number of mol substrate converted per enzyme active site per minute since an enzyme molecule may contain more than one active site. If the number of active sites per mol of enzyme is also known, the activity may be expressed as the catalytic centre activity, which corresponds to mol product formed, or substrate used, per min per mol active site (catalytic centre). The term ‘turnover number’ has also been used, but there is no clear agreement in the literature as to whether this refers to the molecular or the catalytic centre activity. Although the Unit of enzyme activity, and the quantities derived from it, are most widely used, the Katal (abbreviated to kat) is favoured by some chemists. This has the second, rather than the minute, as the unit of time, in conformity with the International System of units (S.I. Units). One Katal corresponds to the conversion of 1 mol of substrate per second. The relationships between Katals and Units are:

1 kat = 60 mol·min^−1^ = 6 × 107 Units (3)

1 Unit = 1 μmol·min^−1^ = 16.67 nkat (4)

It is important to specify the conditions used for enzyme activity determinations, since activity is affected by factors such as pH, temperature, ionic strength and substrate concentration. Temperatures of either 25 or 30 °C have often been used as standards for comparative purposes and either one is preferable to the ill-defined “room temperature”, but it may be appropriate to use a more physiological temperature. There is no clear recommendation as to pH and substrate concentration except that these should be stated. Again, it may be appropriate to use physiological pH values, which may differ from the optimum pH for the reaction, if the results are to be related to the behavior of the enzyme in vivo. Since the activities of some enzymes are profoundly affected by the buffer used and by the ionic strength of the assay mixture, the full composition of the assay mixture must be specified. Guidelines for reporting enzyme data have recently formulated [139] and many journals recommend that these are followed in publications.

Although the rate of reaction is usually proportional to enzyme concentration, it is important to check to ensure that this is so, since there are some situations where this is not so. If the graph of velocity against enzyme concentration does not pass through the origin (a finite value of v at zero enzyme concentration) the most common cause is the failure to subtract a rate or apparent rate of reaction (the blank-rate) that occurs in the absence of the enzyme.

### 3.5. Inhibition

Enzyme inhibitors may act by combining with the enzyme either reversibly or irreversibly. as summarized in Table 3. It is important to distinguish between these types for any interpretation of their behavior, either in vivo or in vitro. This can most simply be done by determining whether dilution, gel-filtration or dialysis results in reversal of the inhibition.

For reversible inhibition, the full inhibition is usually obtained extremely rapidly since there is no chemical reaction involved; simply a noncovalent interaction, where the dissociation constant for the reaction (*k*_−1_/*k*_+1_), is defined as the inhibitor constant, *K*_i_. Inhibition is reversed by dialysis or gel-filtration of the enzyme–inhibitor mixture or, simply by dilution to lower the concentration of the inhibitor. In vivo, the rate of recovery from the effects of a reversible inhibitor will be governed by the rate that it is removed from the tissues by metabolism and elimination.

There are several basic types of reversible inhibitor. For a simple single-substrate reaction the possible modes of inhibitor binding are shown in Scheme 1. They are normally distinguished by their effects on the Michaelis-Menten relationship:

v = *V*_max_ [S]/*K*_m_ + [S] (5)

where *V*_max_ = *k*_cat_ [E] and is the maximum, or limiting, velocity at the enzyme concentration [E] used, [S] is the substrate concentration, and *K*_m_ is the Michaelis constant, which corresponds to the substrate concentration that gives half maximum velocity. The kinetic equations describing the different types of reversible inhibition are treated in most biochemistry textbooks and their behavior is summarized in Table 4.

The reciprocal rearrangement of the Michaelis–Menten equation, to give a linear dependence of 1/*v* upon 1/[S], often known as the Lineweaver–Burk or double-reciprocal plot, has commonly been used. It is useful for displaying the differences between the kinetic behavior of the different inhibitor types, but it is an extremely inaccurate way of determining kinetic parameters (see [140]) for which a direct, nonlinear regression fit to the Michaelis–Menten equation is to be preferred to any of the linear rearrangements. The behavior in terms of the double-reciprocal plot is shown in Table 4, since that representation has been used in many publications.

#### 3.5.1. Competitive Inhibitors

**Scheme 2:**
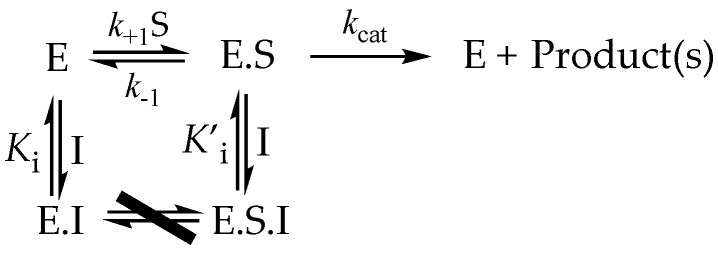
Simple modes of reversible inhibition. The step involving substrate binding to the E.I complex (struck through) is ignored in simple treatments of this system.

**Figure 8:**
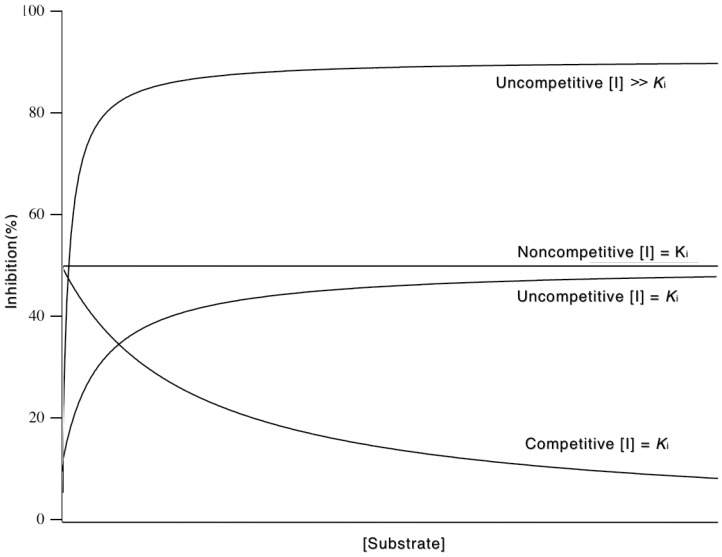
The effects of substrate concentration on the effectiveness of reversible enzyme inhibitors. Values are for the inhibitor concentrations that are shown, relative to their *K*_i_ values.

Competitive inhibitors, whose binding is indicated by the inhibitor constant *K*_i_ in Scheme 2, bind to the same site on the enzyme as the substrate; substrate analogues and products are often competitive inhibitors. The enzyme can either bind substrate or inhibitor but not both at once. At very high substrate concentrations the inhibitor will be displaced from the enzyme; therefore, *V*_max_ is unchanged, but in the presence of inhibitors more substrate will be needed to get to *V*_max_. Thus, more substrate is also needed to reach *V*_max_/2. So, for competitive inhibitors, *V*_max_ is unchanged but *K*_m_ is increased as shown in Table 4. Thus, at any fixed inhibitor concentration, the degree of inhibition will decrease as the substrate concentration is increased, tending to zero as [S] becomes very large, as illustrated in Figure 8.

The simple treatment of competitive inhibition implies the inhibitor binding to the same site as the substrate. However, it is possible to envisage cases where the inhibitor binds to an adjacent site that prevents substrate binding whilst substrate also prevents inhibitor binding. Such a situation appears to occur with edrophonium (*N*-ethyl-3-hydroxy-*N*,*N*-dimethylanilinium), which competitively inhibits AChE by binding to the PAS [141]. An allosteric effect, whereby inhibitor binding results in a conformational change which prevents substrate binding, whereas substrate binding causes a conformational change that prevents inhibitor binding, is also possible, as appears to be case for several AChE inhibitors [142].

#### 3.5.2. Uncompetitive Inhibitors

Uncompetitive inhibitors, represented by the inhibitor constant *K*’_i_ in Scheme 2, do not bind to the free enzyme but only to an enzyme–substrate complex. As the inhibitor concentration is increased more and more enzyme will be converted to the, unproductive, E.S.I form. Thus, both the *K*_m_ and the *V*_max_ values are decreased by the same amount, as shown in Table 4, and at any fixed inhibitor concentration, inhibition will increase towards a maximum value as the concentration of the S is increased (Figure 8, [143]); this is the exact opposite of the competitive case.

#### 3.5.3. Mixed and Noncompetitive Inhibition

This represents the case where the inhibitor can bind both to the free enzyme (competitive) and to the enzyme-substrate complex (uncompetitive). Such a compound would behave as a mixture of a competitive and an uncompetitive inhibitor; hence the term mixed inhibition. The competitive effect (governed by *K*_i_ in Scheme 2) will increase the *K*_m_, whereas the uncompetitive effect (governed by *K*’_i_ will decrease both *K*_m_, and *V*_max_. The net result of these effects will depend on the relative values of *K*_i_ and *K*’_i_.

The value of *K*_m_ will be increased if the inhibitor has a higher affinity for the free enzyme than for the E.S complex (*K*_i_ < *K*’_i_), whereas in the converse case (*K*_i_ > *K*’_i_) *K*_m_ will be decreased. *V*_max_ will be decreased in all cases, as shown in Table 4. In the limiting case, where the inhibitor binds equally well to E and E.S (*K*_i_ = *K*’_i_) the increase in *K*_m_ owing to the competitive effect will be exactly balanced by the decrease resulting from the uncompetitive element. Thus, in this case *V*_max_ will be decreased and *K*_m_ unaffected. This has often been termed as noncompetitive inhibition but, confusingly, some authors use this term to cover mixed inhibition as well on the spurious grounds that it is difficult to distinguish between these two types of inhibition. Whereas this may be the case if the, inaccurate, double-reciprocal plot was used, that should not be the case with more accurate curve-fitting procedures. Sometimes the term *true noncompetitive inhibition* is used to avoid ambiguity.

### 3.6. More Complex Reversible Inhibitor Behavior

The mixed and noncompetitive mechanisms discussed above may appear somewhat oversimplified since, if the inhibitor can bind to both E and E.S, it would be reasonable to suppose that substrate might also bind to the enzyme-inhibitor complex, resulting in a scheme that includes the step that is struck-through in Scheme 1. If this step is included, the inhibitor binding-steps, which were simple equilibria because they formed dead-end complexes that could not react further, become more complex. If it is assumed that the rates of dissociation from the inhibitor- and substrate-containing complexes are so rapid that they remain at thermodynamic equilibrium, the kinetic behavior will be identical to that given by in the mixed and noncompetitive cases. However, under steady-state conditions the complex situation may result in the behavior departing from that predicted by the Michaelis-Menten equation (see [27,143,144]).

#### 3.6.1. Partial Inhibition

**Scheme 3:**
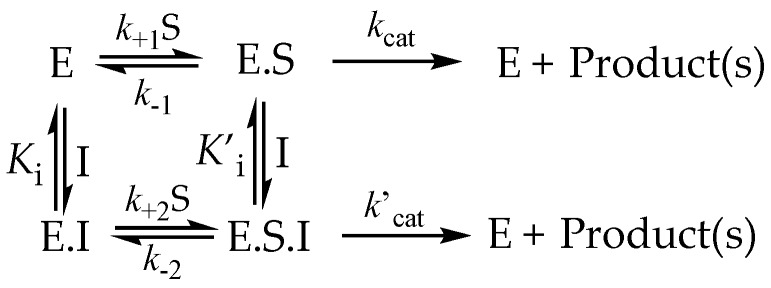
Partial inhibition.

An inhibitor may not completely prevent the reaction from occurring. For example, the binding of a competitive inhibitor may raise the apparent *K*_m_ for the substrate without completely preventing its binding, whereas partial noncompetitive inhibition would produce a new SES species that was less active than ES, reducing the apparent *k*_cat_ to a finite, non-zero, value. A general mechanism for such partial inhibition can be written as shown in Scheme 3. The kinetic behavior of such systems, which can be quite complex, has been presented elsewhere [134,143].

#### 3.6.2. Tight-Binding Inhibitors

Some inhibitors bind so tightly to an enzyme that inhibition occurs with inhibitor concentrations that are comparable to those of the enzyme. In such cases, the rate of onset of inhibition, which will be governed by the second-order rate constant for combination between E and I, can be relatively slow. Conversely the rate of dissociation after dilution of the enzyme-inhibitor complex would also be expected to be slow. The kinetic equations governing such inhibition have been presented [144] and graphical [145] and computational [146,147] methods have been reported for determining the type of inhibition and *K*_i_ values. Inhibitors of MAO that have been shown to behave in this way include cimoxatone [148], harmaline (see [149]) and methylene blue [150].

#### 3.6.3. High-Substrate Inhibition

**Scheme 4:**
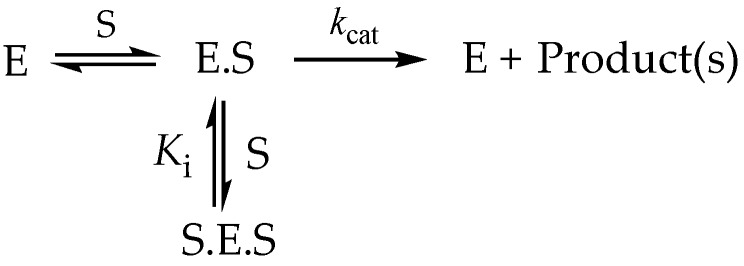
Inhibition by high substrate concentrations.

As discussed above, AChE is inhibited by high concentrations of substrate, which has been attributed to substrate binding to the PAS as well as to the active site. Such high substrate inhibition may be most simply represented by the model shown in Scheme 4, where a second molecule of substrate binds to the ES complex. This gives a relationship of the form:

(6) $$v=\frac{V_{max}\;\left[S\right]}{K_{m}+\left[S\right]+\frac{{\left[S\right]}^{2}}{K_{i}}}$$

**Figure 9:**
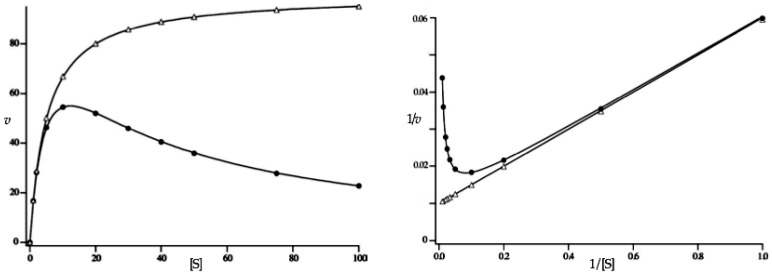
Enzyme inhibition by high substrate concentrations. The illustrative curves shown were calculated for a *K*_m_ value of 5 and a *K*_i_ value of 30 with *V*_max_ = 10, all in arbitrary units.

Thus, the velocity will increase in the normal way as substrate concentration is increased, but then decline at higher concentrations, as the term [S]^2^/*K*_i_ becomes dominant, leading to a plot such as those shown in Figure 9. Such behavior may restrict the range of substrate concentrations that can be used for determining *K*_m_ and *V*_max_ values. The treatment of high-substrate inhibition data has been discussed in detail elsewhere (see [134,143]). If such inhibition is observed, it is necessary to carry out appropriate controls to ensure that it is a property of the enzyme and its substrate rather than an artefact arising from failure to control the pH, ionic strength or dielectric of the assay medium correctly. It is also necessary to show that the inhibition is due to the substrate itself rather than to an inhibitory contaminant. Such contamination has been found to affect assays when the free-base form of benzylamine, obtained from some sources was used for MAO assays rather than benzylamine hydrochloride [135]. High-substrate inhibition, which is time-dependent, has also been reported for MAO-B from rat liver with 2-phenylethylamine as substrate [151].

#### 3.6.4. Reactions Involving More than One Substrate

Most enzyme catalyzed reactions involve reactions between two or more compounds. In the case of the ChEs, there are two substrates, the ester and water. However, since water is present at very high concentrations (about 55.5 M in pure water) and it is rather difficult to vary its concentration, it is generally assumed that it can be ignored. The MAO-catalysed reaction also involves oxygen. The type of inhibition given towards the amine substrate will not be the same with respect to oxygen. The kinetic behavior observed in such cases can be used to show how the reaction proceeds. This has been the subject of several detailed studies involving MAO (see [144]).

Most enzyme catalyzed reactions involve reactions between two or more compounds. In the case of the ChEs, there are two substrates, the ester and water. However, since water is present at very high concentrations (about 55.5 M in pure water) and it is rather difficult to vary its concentration, it is generally assumed that it can be ignored. The MAO-catalysed reaction also involves oxygen. The type of inhibition given towards the amine substrate will not be the same with respect to oxygen. The kinetic behavior observed in such cases can be used to show how the reaction proceeds. This has been the subject of several detailed studies involving MAO (see [144]).

### 3.7. Competition Between Substrates

**Scheme 5:**
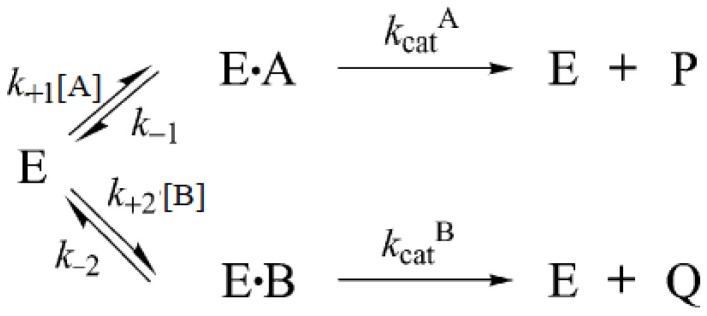
Competing substrates.

Many enzymes may be exposed to more than one substrate at the same time. When two substrates compete for the same enzyme, the situation can be represented as shown in Scheme 5.

In this case, each substrate will act as a competitive inhibitor of the other. The rate of formation of P (*v*_a_) will be:

*v*_a_ = *V*^A^_max_ [A]/*K*^A^_m_(1 + [B]/*K*^B^_m_) (7)

and the rate of formation of Q (*v*_b_) will be:

*v*_b_ = *V*^B^_max_ [B]/*K*^B^_m_(1 + [A]/*K*^A^_m_) (8)

where *K*^A^_m_ and *K*^B^_m_ are the *K*_m_ values for substrates A and B, respectively, and *V*^A^_max_ and *V*^B^_max_ are the maximum velocities in the absence of the other substrate.

When the two substrates are both present, the relative rates of reaction will be given by:

*v*_a_/*v*_b_ = (*V*^A^_max_ [A]/*K*^B^_m_)/(*V*^B^_max_ [B]/*K*^A^_m_) (9)

In this case *k*_cat_ values can replace the corresponding *V*_max_ values since [E] is the same for both substrates and *k*_cat_ = *V*_max_/[E] Thus, the relative rates will be determined by their relative *k*_cat_/*K*_m_ (or *V*_max_/*K*_m_) values and the substrate concentrations. If each substrate is present at the concentration corresponding to its *K*_m_ value this simplifies to *v*_a_/*v*_b_ = *k*^A^_cat_/*k*^B^_cat_. In the case of MAO this relationship has also been used show whether the same enzyme is involved in the metabolism of two different substrates [152].

### 3.8. Irreversible Inhibition

The simplest type of irreversible inhibition involves direct reaction with a group or groups on the enzyme to form a stable, covalently modified enzyme. Such compounds are usually not specific for any particular enzyme but will react with amino acid side-chains having similar reactivity in many different enzymes and often with several different residues in the same enzyme. The reaction is a time-dependent and not reversible. The behavior and uses of such indiscriminate inhibitors has been discussed in detail elsewhere [143] but is of little concern in the present context.

**Figure 10:**
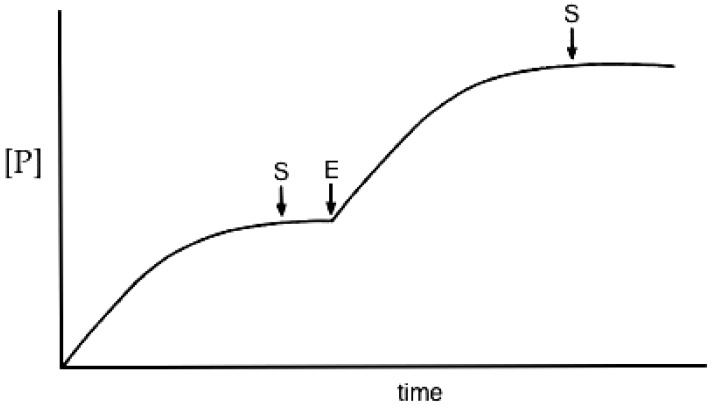
Time-course of an enzyme-catalysed reaction in the presence of an irreversible inhibitor. The inhibitor concentration in this example is sufficient to cause complete inhibition.

**Scheme 6:**
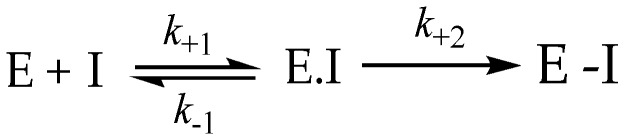
Irreversible active site directed inhibition.

In the presence of an irreversible inhibitor the rate of product formation should steadily decrease until it ceases, as shown in Figure 10. Addition of more substrate should have no effect, but addition of more enzyme should restart the process. Specific irreversible inhibitors form an initial noncovalent complex with the enzyme, analogous to the enzyme-substrate complex and then react within that complex to give the irreversibly inhibited species. The need for initial inhibitor binding confers specificity on the process. There are two types of specific irreversible inhibitor. In one case the inhibitor contains a chemically reactive group attached to a substrate analogue. The high local concentrations of that group and a group on the enzyme surface within the noncovalent enzyme–inhibitor complex will lead to chemical reaction between them, resulting in irreversible inhibition. Inhibitors of this type are often known as active site directed inhibitors (ASDINS). Their behavior is represented by the mechanism in Scheme 6 where E-I is the irreversibly inhibited species.

In the second type, the inhibitor is not intrinsically reactive. It first forms a noncovalent complex with the active site of the enzyme, and subsequent reaction within that complex leads to the generation of a reactive species that reacts with the enzyme to form the irreversibly inhibited species. Inhibitors of this type are known as mechanism-based, *k*_cat_ or suicide inhibitors. They can show a higher specificity towards a target enzyme because the generation of the effective inhibitory species from an essentially unreactive compound involves part of the catalytic function of the enzyme itself. Furthermore, the lack of intrinsic reactivity minimizes the possibility of unwanted reactions with other tissue components.

It is possible to characterize this system in terms of two parameters, *K*’_i_ (sometimes designated K_I_), which is the inhibitor concentration that gives half maximal rate of inhibition and *k*_+2_ (or *k_inact_*) from analysis of rates at which activity is lost at different inhibitor concentrations [153]. This procedure has been used to characterize the inhibition of MAO by the acetylenic inhibitors clorgyline, *l*-deprenyl and pargyline [154] and the inhibition of AChE by some organophosphates and carbamates [155].

### 3.9. Irreversible Inhibitors as Substrates

**Scheme 7:**
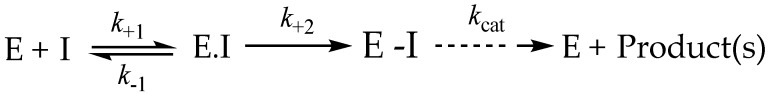
Reaction of inhibitors that act as poor substrates

Since such inhibitors mimic the behavior of substrates, it is not surprising that some compounds in this class are in fact very poor substrates and the activity slowly recovers as the reaction proceeds (Scheme 7).

If *k*_+2_ is sufficiently slow inhibition will be time-dependent, but it will be slowly reversed by dialysis. The inhibition of AChE by rivastigmine (see above) has been accounted for by this model, in which the hydrolytic step is extremely slow [156].

**Scheme 8:**
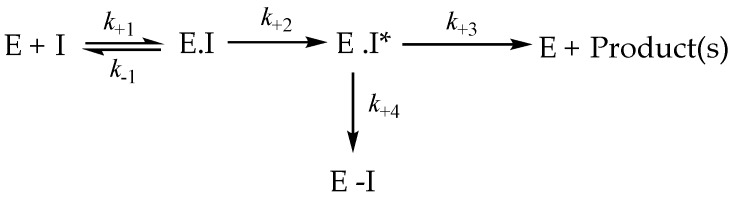
Reactions of a suicide substrate.

In other cases the enzyme-catalysed formation of product and the irreversible inhibition can be competing processes in which the enzyme inhibitor complex (E.I) is converted to an activated intermediate (EI *) which can either react to form product(s) or to form an irreversibly inhibited species (E-I) as shown in Scheme 8. Such inhibitors are often termed *suicide substrates*.

The hydrolysis and ‘ageing’ process that can occur in the interactions of AChE with organophosphates, discussed above, and the interaction of MAO with a number of inhibitory substrates, including the pro-neurotoxin MPTP (1-methyl-4-phenyl-1,2,3,6-tetrahydropyridine) [157], the anticonvulsant milacemide [158] and the never marketed MAO-B inhibitor MD 780236 [159] are examples of this behavior. This process can be characterized by two parameters, *K*’_i_ (the inhibitor concentration giving half maximal rate of product formation or inhibition) and an overall rate constant rate constant *k*_inact_, where:

*k*_inact_ = *k*_+2_*k*_+4_/(*k*_+2_ + *k*_+3_ + *k*_+4_) (10)

Procedures for analyzing such systems have been developed [160,161]. An important additional parameter that can also be obtained is the partition ratio (r), defined as

*r* = *k*_+3_/*k*_+4_ or *r* = [*p*_∞_]/[E] (11)

where [*p*_∞_] is the concentration of product when all reaction is finished, Thus, r is the number of mol product produced per mol of enzyme at complete inhibition. Studies on the inhibition of MAO-B by the anticonvulsant milacemide and the neurotoxin MPTP have shown that this is dependent on the species from which the enzyme was obtained [162,163].

### 3.10. IC_50_ Values

The IC_50_ (IC-50 or I50), which corresponds to the inhibitor concentration required to give 50% inhibition is often used as a means of expressing inhibitor potency. However, this can be misleading since it is dependent on the type of inhibition involved. In the case of reversible inhibitors, the IC_50_ will depend on the type of inhibition and the substrate concentration.

These relationships are summarized in Table 5, along with the corresponding percentage inhibition values. These relationships show that only in the case of true noncompetitive inhibition will IC_50_ be independent of the substrate concentration. In the case of an irreversible inhibitor, the IC_50_ value will be time dependent, tending to [E]/2 at longer times for simple irreversible inhibitors. Clearly, the IC_50_ values have little meaning without knowledge of the inhibitory mechanism involved.

## 4. MAO Assays

**Figure 11:**
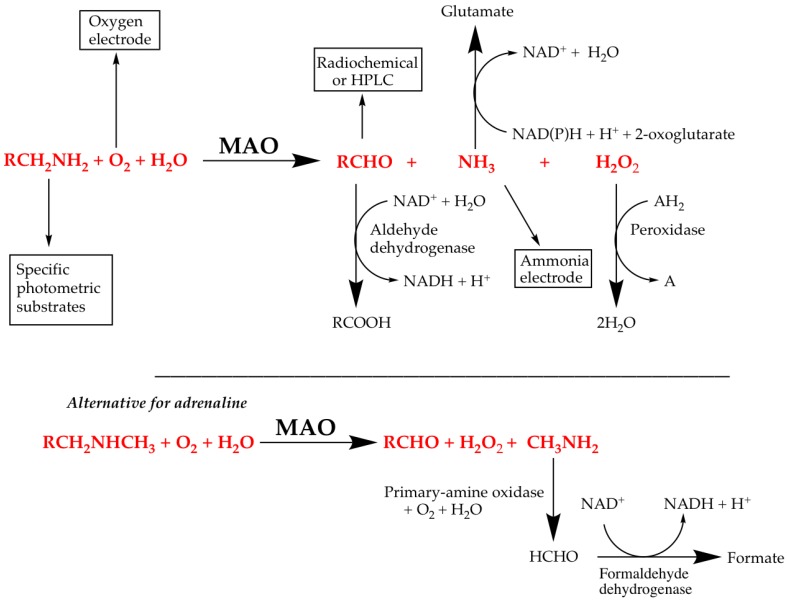
Assaying MAO. The schemes show the reaction catalysed by MAO and how the components have been used to assay MAO activity.

MAO A and B are enzymes with wide specificity for their role in endogenous and xenobiotic metabolism. They oxidize many amines (primary, secondary or some tertiary), providing a multitude of ways to assay the activity [135,164,165,166,167,168,169,170,171,172,173,174]. The reactions that can be used to link MAO catalytic activity to conveniently measurable products are shown in Figure 11. Protocols for most of these assays can be found in [135].

The types of assay are listed in Table 6; the one used will depend on the purpose. Direct and continuous assays are often preferred for kinetic work, but may be less convenient for high throughput screening Direct stopped assays such as those using radiolabelled amines are expensive and inconvenient due to the sample processing required. They also need careful controls to ensure linearity throughout the chosen time-frame and to ensure replicable post-assay processing. A number of assay procedures involve the use artificial substrates and, although these can be valuable for activity comparisons and inhibitor assessment, it must be realized that the kinetic mechanism followed by MAO depends on the substrate used, and, thus, the type of inhibition that is observed may not be the same as that operating with a physiological substrate.

The production of a fluorescent product is in demand for measuring MAO activity not only in vitro but also in living cells. For example, MAO-catalysed oxidation of a propylamine recognition group in the fluorescent skeleton of 1,8-naphthalimide followed by β-elimination releases the fluorophore (4-hydroxy-*N*-butyl-1,8-naphthalimide) that is quantified by ratio of fluorescence intensity at 550 nm and 454 nm, selectively illuminating MAO A in cells [175]. For sensitivity, fluorescent products come second to radiolabelled versions but the convenience and safety of fluorescence methods has resulted in their use in versions of coupled assays for high-through-put screening. This article focuses on the assays used for in vitro assessment of the inhibition of MAO activity. In contrast to spectrophotometric determinations, such as the aldehyde dehydrogenase-coupled assay [see 144], where absorbance is proportional to concentration, fluorescence determinations do not yield quantitative reaction rates unless calibration curves for the product being measured are determined separately.

### 4.1. Direct Assays for MAO Activity

A convenient spectrophotometric method for both MAO A and B measures the absorbance change from the oxidation of kynuramine to 4-hydroxyquinoline at 314 nm (extinction coefficient = 12,100 M^−1^ cm^−1^) [135,164]. Kynuramine is a substrate for both MAO A and MAO B, although kinetic studies on MAO B have usually used benzylamine as the substrate, measuring the change in absorbance at 250 nm (extinction coefficient = 12,800 M^−1^ cm^−1^) [135]. Inhibitors that absorb in the UV can be problematic in this procedure, due to high absorbance.

### 4.2. Coupled Assays for MAO Activity

A recently reported chemically coupled assay that takes advantage of the of the acid-induced conversion of the aldehyde product to a form that induces the aggregation of silole to a fluorescent product has been reported [172]. More convenient are the various enzyme-coupled assays developed for high throughput work, using peroxidase to couple the H_2_O_2_ formed by MAO activity to the oxidation of a dye. Several dyes have been employed for this purpose, but, not surprisingly, those that are available commercially in assay kits, have become the most widely used. The use of 10-acetyl-3,7-dihydroxyphenoxazine (Amplex Red™) that oxidizes to the fluorescent compound, resorufin, with fluorescence detection is sensitive and convenient, but careful controls for interference in the detection components by the compounds to be studied as inhibitors of MAO are essential. For example, a series of disubstituted indolyl thioureas, particularly those with phenolic groups, being assessed as inhibitors of MAO were found to be potent inhibitors of horseradish peroxidase preventing the use of this coupled assay [176]. Furthermore, it is necessary that the substrate used is not oxidized by the H_2_O_2_-peroxidase system or the detection dye. The luminescence assay (MAO-Glo™ [170] minimizes the problem of inhibition of the coupling system by using a luciferin derivative as the substrate incubated with MAO alone, then stopping the reaction by adding excess coupling system. This makes the MAO-Glo method a stopped (single time point) assay so linearity with time must be verified. As with all coupled assays, careful controls are required and it is advisable to validate hits with direct or different assays.

The horseradish peroxidase coupled assay kits, fulfill the requirements for sensitive, continuous, high-throughput assays but must be adapted for medicinal chemistry use in inhibitor assessment. The manufacturer’s protocols and the kits are designed to measure any MAO activity present in a sample, using a fixed substrate concentration above *K_m_*. This is not ideal for comparing reversible inhibition of two enzymes with different *K_m_* values. To determine the IC_50_ of a compound expected to give reversible inhibition, the substrate concentration should be sub-saturating so that in the steady state there is free enzyme available for binding either inhibitor or substrate. For medicinal chemists investigating the selectivity of their compounds for MAO A or MAO B, the proportion of free enzyme should be the same for both enzymes. In practice this means using the substrate at around 2 × *K_m_*, remembering that the two enzymes have difference substrate specificities (Table 1).

### 4.3. Controls for Coupled Fluorescence Assays of MAO Activity

All such coupled assays require similar controls and the following account will summarize them in terms of the, most commonly used, Amplex Red procedure. Before screening any new series of compounds for inhibition of MAO, the assay conditions must be optimized and tested for interference by those particular compounds.

**Figure 12:**
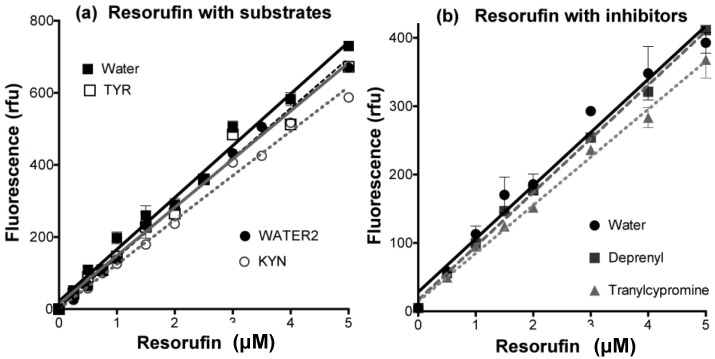
Quenching of resorufin by common inhibitors and substrates. The relative fluorescence of resorufin alone or (**a**) with tyramine or kynuramine; (**b**) with inhibitors.

1. Determine any effect of the new compounds on resorufin fluorescence. Measure the fluorescence of fresh solutions of resorufin at 0–5 μM (excitation at 535 nm, emission at 595 nm) in the absence (solvent alone) or presence of any chemical compound at the highest concentration to be tested. Quenching of fluorescence by amines and inhibitors of MAO is frequently observed, even (slightly) for routine substrates or inhibitors (Figure 12).
2. Check that the new compounds do not inhibit the coupling enzyme, horseradish peroxidase (HRP). The assay mixture in a final volume of 200 μL 50 mM potassium phosphate buffer (pH 7.4) should contain: H_2_O_2_ (50 μM), Amplex Red (200 μM), HRP (0.02 U/mL) and a single compound of interest or its solvent as the control. The HRP activity is determined by the fluorescence of the resorufin formed with time at 30 °C [176].
3. Check the linearity of product generation with enzyme and time, and verify that less than 10% of substrate is consumed.
4. When comparing two enzymes, determine the *K*_m_ for substrate to ensure that the substrate concentrations used in the inhibitor screen gives similar enzyme saturation for each enzyme.
5. If the inhibition increases with time, check whether it is reversible (and, for a full investigation, determine the mechanism).
6. If the inhibition is irreversible, the IC_50_ value will depend on time. This should be checked by preincubating the enzyme and inhibitor for various times before the addition of substrate to measure the activity remaining.
7. Some irreversible inhibitors of MAO are substrates with high partition ratios, so can generate H_2_O_2_ in the absence of the normal substrate (for example, phenelzine [177]). This further detracts from the meaning of IC_50_ results.

With the assay conditions verified, new compounds can be screened with confidence.

### 4.4. Optimized Conditions for MAO Inhibition Screening Using the Amplex Red Coupled Assay

Providing *all* the control checks are done, the Amplex Red assay is convenient for high-throughput screening of novel compounds in medicinal chemistry. However, the conditions that must be used are different from the standard kit protocol. The kit assay is designed to detect the total activity of MAO present, so it uses a high substrate concentration (1 mM tyramine). For reversible inhibitor assessment (most compounds in recent articles are reversible inhibitors), the substrate concentration must be sub-saturating. Using commercial membrane-bound human MAO, the *K*_m_ values for tyramine are different for MAO A (0.4 mM) and MAO B (0.2 mM) (see Table 1). At 1 mM tyramine, about 70% of MAO A active sites will be occupied by substrate, in contrast to 83% for MAO B. Using 2 × *K*_m_ as the substrate concentration in routine screening assays for reversible inhibitors gives satisfactory rates of reaction with a defined saturation of the active sites (67%).

**Figure 13:**
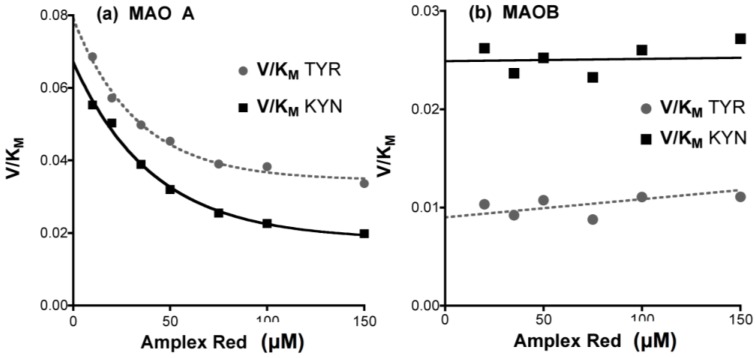
Amplex Red inhibits MAO A but not MAO B. (**a**) The values of V/Km decrease for MAO A as Amplex Red increases. (**b**) The values of V/Km for MAO B are close to constant, as is desirable for a screening assay.

A further complication is that Amplex Red inhibits MAO A (Figure 13) so the concentration should be 50 μM in the well. Alternatively, to set a best common condition for both MAO A and B, 100 μM could be used. Amplex Red and resorufin have a tricyclic structure, like that of Methylene Blue (Figure 1) that binds tightly to MAO A (*K*_i_ = 27 nM) but poorly to MAO B (*K*_i_ = 5 μM) [150].

### 4.5. Species Differences in Inhibition of MAO

The differences in MAO A and B sequence [178] and overall structure [18] are small, but the minor differences in the active site mean that MAO A and MAO B have different catalytic efficiencies [19]. Not surprisingly, MAO enzymes from different species have different ligand affinities [162,179,180]. Other examples include the 10,000-fold difference between the *K*_i_ values for oxazolidinone inhibition of MAO B from human versus bovine liver [181] and smaller differences between rat and human MAO IC_50_ values that have been reported for various inhibitors [179,181,182]. A comparison of the IC_50_ values obtained for the four enzymes inhibited by ASS234, a multi-target compound designed for Alzheimer’s disease, in commercial human enzymes versus the more economical alternative sources is shown in Table 7.

## 5. Determination of Acetylcholinesterase Activity

**Figure 14:**
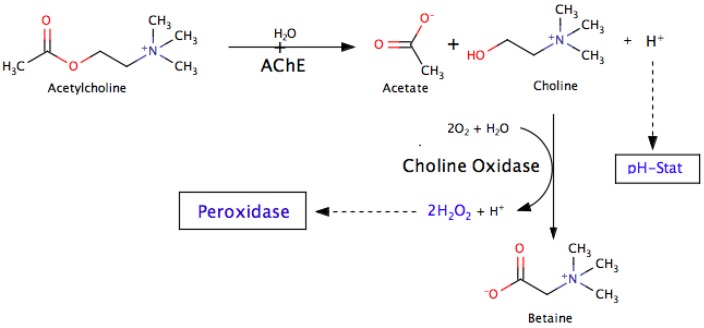
Hydrolysis of acetylcholine. The products used as the basis of assay methods are indicated.

Numerous assay procedures have been reported for the cholinesterases, although many of these are modifications of earlier methods to make them more appropriate for specific applications, such as large-scale screening for inhibitors. Before considering the procedures that that may be used, it is necessary to consider the applications for which assays are desired. At the basic level, they might be used to answer the simple questions of whether an individual has been exposed to a toxic AChE inhibitor or whether an area is contaminated with such compounds that may have been used as pesticides or herbicides. Such assays are sometimes carried out in the field where elaborate laboratory equipment is not available and a high degree of accuracy is not required. The details of such methods have been covered in excellent reviews [136,185,186]. More sophisticated procedures are necessary for studies on the mechanisms of inhibition and the kinetic constants involved. This account will concentrate on assays (such as shown in Figure 14) used for accurate kinetic purposes and refer only briefly to the others.

### 5.1. Radiochemical Assays

Radiochemical assays used in the past were based on the release of acetate from on acetylcholine labelled in the acetyl moiety with ^14^C or ^3^H. The reaction could be stopped, e.g., by acidification, and the labelled acetate extracted into an organic solvent-based scintillant for counting. Alternatively, the excess acetylcholine may be removed by precipitation with reineckate before counting (see [187]). Separation by ion-exchange chromatography or HPLC has also been used. The radiochemical method had the advantage of using the natural AChE substrate rather than a synthetic analogue. Although simple and highly sensitive, its use has become restricted on health and safety grounds.

### 5.2. Hydrogen Ion Liberation

As illustrated in Figure 14, the hydrolysis of acetylcholine liberates a hydrogen ion. Thus, the reaction can simply be followed by determining the pH change. Originally, the method involved just two measurements at different times, with the activity being then expressed as ΔpH/hour [188]. A variation involved measuring the time taken for the pH to fall by a set amount. A more elaborate development of this approach for screening acetylcholinesterase inhibitors has been to immobilize the enzyme on to the surface of the pH electrode [189]. However, generally one would wish the pH to remain constant during the reaction, and furthermore, the pH scale is logarithmic rather than linear. This has been overcome titrating with an alkaline solution to keep the pH constant and measuring the amount added with time. Originally this was done manually but this has been superseded but the pH-stat (see [190]), which automatically adds dilute alkali (NaOH or KOH) to the assay mixture to keep the pH constant whilst recording the rate of addition to give a direct measure of the reaction rate. An alternative procedure involves the use of a pH indicator to provide a colorimetric determination of the pH change (see [191]). A variety of indicators, such as phenol red, have been used.

A difficulty with all such methods is that the assay mixture must be unbuffered, or only weakly buffered. For example, the protein in serum or blood samples can militate against any pH changes. Furthermore, it may affect the choice of substrate and any other reaction component under test. For example, acetylthiocholine, which is a commonly-used artificial substrate for AChE, is hydrolysed to the weak acid thiocholine that consumes some of the H^+^ released [190]. Although pH methods have been largely replaced by other approaches for the accurate determination of AChE activity, rapid assessments of inhibition can be made by using test-strips comprising entrapped AChE and a suitable indicator (see [192]).

### 5.3. Assays Based on Artificial Substrates

**Figure 15:**
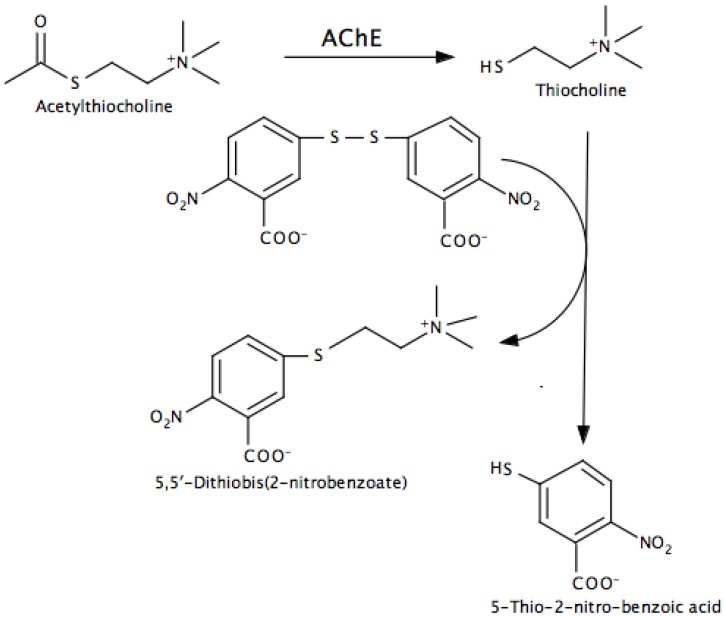
Assay of acetylcholinesterase activity towards acetylthiocholine by the method of Ellman et al. [193].

The most commonly used assay is that devised by Ellman and co-workers [193] that replaces acetylcholine with acetylthiocholine. Hydrolysis by AChE releases thiocholine which can react non-enzymically with the thiol reagent 5,5’-dithiobis-2-nitrobenzoic acid (DTNB) to release the bright yellow product 5-thio-2-nitrobenzoic acid (TNB) ion, as illustrated in Figure 15. Since DTNB does not interfere with AChE activity the reaction can be performed in a single vessel containing buffer AChE, DTNB and acetylthiocholine. The reaction with DTNB is sufficiently fast for the AChE reaction to be limiting so that the development of the yellow colour, measured at 412 nm, provides an accurate measure of the rate of acetylthiocholine production. Unfortunately, the extinction coefficient was originally underestimated, as 13,600 M^−1^ cm^−1^ and so several reports on the enzyme activity were incorrect. This may have been because the absorbance coefficient is dependent on temperature as well as pH. The values in 0.1M phosphate buffer, pH 7.4 at 412 nm are 14,500 M^−1^ cm^−1^ at 25 °C and 13,800 M^−1^ cm^−1^ at 37 °C. These and the values at other temperatures and wavelengths have been tabulated by Eyer et al. [194]. Variations in which the reaction is stopped after a fixed time and the absorbance is then determined have been used for high-throughput plate reader (see [195]) or robotic automated assays [196].

Clearly account must be taken of the possibility that other reaction components might interfere the detection system. Since the hemoglobin in blood samples absorbs significantly at 412 nm, the higher wavelength of 436 nm, which reduces the TNB absorbance by about 20%, has been used [197]. The presence of compounds that are capable of reducing the disulfide bond in DTNB may result in undesirably high blank rates, so that the controls in which thiocholine is omitted must be used. The thiol groups in the tissue samples such as those in glutathione and proteins (for example, hemoglobin, and albumin) react with DTNB at differing rates, and some procedures recommend incubating the tissue sample with DTNB for a fixed time before the addition of substrate. However, it is preferable to wait until there is no change in absorbance before doing this. Some oximes may also interfere with this assay procedure [198]. However, with appropriate experimental design the Ellman procedure has proven to be a robust and accurate method for studying cholinesterase activity for a variety of purposes (see [199]).

Acetylthiocholine is also a substrate for BChE, so BChE inhibitors may be added in order to study AChE activity in the presence of BChE. Butyrylthiocholine is not a substrate for AChE so can be used to determine BChE activity alone (see [200]).

Several other substrates have been developed for cholinesterase assays, including the *p*- and *o*-nitrophenyl acetate and butyrate esters, which liberate the yellow coloured nitrophenol on hydrolysis [201]. However, these are substrates for other tissue enzymes, so they have low specificity. Indoxylacetate has been used as a convenient substrate, since the indole released on hydrolysis is spontaneously oxidized to the coloured compound indigo blue [see [202]. However, this oxidation reaction is complex [203] and may not be sufficiently rapid under all conditions. Indeed, the immediate products of indole oxidation, indoxyl and indigo white, formed *en route*, have been used for absorbance and fluorescence determination of cholinesterase activity [204,205]. Other synthetic substrates that have been used include 2,6-dichloroindophenol acetate [206] and resorufin butyrate [204].

### 5.4. Enzyme Coupled Assays

An assay commonly in use involves the use of two coupling enzymes, choline oxidase to convert choline to betaine +H_2_O_2_ (Figure 14) and peroxidase to couple the H_2_O_2_ to the oxidation of a *leuko*-dye, as discussed in connection with the assay of MAO. Several different oxidizable dyes have been used in the peroxidase reaction including 4-aminoantipyrine, for spectrophotometric assay [207], luminol, for luminescence assays [208], and *N*-acetyl-3,7-dihydroxyphenoxazine (Amplex Red^TM^) for fluorescence assays [209]. Amplex Red has proven to be the most widely used of these, because assay kits based on this method are available from a number of suppliers. The controls and precautions necessary are similar to those discussed in connection of MAO assays with this compound, with the additional complication that there are two coupling enzymes, neither of which should be rate-limiting or be affected by any inhibitor, or other reaction component, under test.

## 6. Virtual Screening

Rational design of ligands has been greatly facilitated by the availability of crystal structures of target enzymes. Docking programmes allow basic visualization of ligands in the active site and prediction of suitable modifications to small molecules to improve affinity and selectivity. Virtual screening using scoring functions to estimate binding energies for each compound can be used to seek new ligands from large chemical libraries. Probabilistic models, based on the compound structure, have also been used to predict both the primary pharmaceutical target and any off-target interactions [4,6]. Although virtual screening has proved successful in identifying new leads, the accuracy of predicted binding energies is doubtful [210]. Docking methods have become major tools in rational drug design, particularly for multi-target compounds, to probe the effects of alterations in ligand structure on the affinity of the compound for the target enzyme(s). However, predictions of the functional effect of ligand binding on the catalysis by the target(s) still requires verification by assays. Docking cannot predict the efficiency of the mechanism-based inhibitor—only its initial reversible binding. Combining information from theoretical and laboratory assays facilitates the design of analogs from a lead compound, as exemplified by multi-potent ASS234 analogs [183]. In another example, experimental values closely paralleled the predicted values in a test set of carbamate compounds when 3D-Quantitative-Structure-Activity Relationship (3D-QSAR) was used to define 3D-pharmacophores for inhibition of MAO A and B, and of the ChEs [211].

The application of computational approaches to MTDL design has been reviewed recently [4]. The monoaminergic neurotransmitters interact with multiple binding proteins, such as MAO, COMT and the families of specific receptors and transporters. The breakdown enzymes such as MAO can be designated as promiscuous targets binding many compounds while compounds that bind to both MAO and to receptors are promiscuous ligands. When multiple moieties for binding to different targets are incorporated into MTDL, investigation for off-target activity is essential, particularly for the monoamine system where each receptor can have a different effect.

## 7. Inhibition for Effective Drugs

Some of the nerve gases, suicide inhibitors of acetylcholinesterase, discussed in Section 2.4, cholinesterases have been used therapeutically but they are also toxins. Their off-target interactions with the neurotoxin-target esterase can cause further deleterious effects. Since carbamates do not affect NTE, they have proven to be safer alternatives. Many of these are behave as poor substrates for AChE, in which the rate of hydrolysis of the carbamyl-serine intermediate is relatively slow. Their effective half-lives in vivo will depend on the rate of this hydrolytic step. In contrast, the rate of recovery from the effects of reversible inhibitors will be determined by their rates of clearance by metabolism and excretion. The toxicity of irreversible AChE inhibitors, led to the application of reversible and competitive inhibitors for use in AD. The main ones, now in general use, donepezil, galantamine and rivastigmine, are different in structure and also in their target binding sites. Their reported effectiveness in mild-to-moderate AD, leaves open the question of whether, or not, binding to the PAS, rather than, or as well as, to the active site, confers any particular benefit. Despite this uncertainty, one strategy in designing AChE inhibitors for use in against Alzheimer’s disease, has been to incorporate binding both at the catalytic site (to spare acetylcholine) and at the peripheral site (to prevent facilitation of Aβ aggregation). Binding of an inhibitor to two sites on the target can also result in a considerable enhancement of affinity as a result of the chelate effect [212]. Examples of multiple points of binding resulting in increased affinity can be seen, for example, in [213,214,215,216]. Since galantamine and rivastigmine also inhibit BChE, whereas donepezil does not, further evidence would be necessary, before it would be possible to evaluate the possible benefits of inhibiting that enzyme. Each inhibitor has a relatively short duration of action and rivastigmine has been reported to give about 30% occupancy [217].

Many different MAO inhibitors have been used therapeutically and this has resulted in an accumulated understanding of their interactions and metabolism. The enzyme has an important role in the metabolism of ingested and xenobiotic amines in addition to neurotransmitter metabolism. The antidepressant moclobemide, a reversible competitive inhibitor that is selective for MAO A, does not completely prevent the intestinal enzyme from catalysing the oxidation of tyramine. In contrast, irreversible inhibitors that are selective for MAO A, such as clorgyline and those that are non-selective, such as, pargyline, allow tyramine to pass unchanged through the intestinal wall and into the serum, with a consequent vascular crisis [53]. In the brain, moclobemide, provides anti-depressant effects at 74% occupancy of MAO A [218]. The proportion of enzyme molecules occupied by such a reversible inhibitor will vary depending on the local concentration of other ligands, such as the substrates, competing for the active site. With competitive inhibitors, the inhibitor can be displaced from the active site by higher substrate concentrations, as discussed above for intestinal MAO, where the inhibitor may be displaced by dietary tyramine allowing some metabolism to occur [219]. For enzymes such as AChE catalyzing essential processes, using a competitive inhibitor ensures that the metabolism can take place when the substrate spikes in concentration. It should be noted that the amelioration of inhibitory effects by rising substrate concentrations requires a competitive inhibitor, since the effects of a true noncompetitive inhibitor would be unaffected whereas those of an uncompetitive inhibitor would be strengthened.

The early MAO inhibitors used as antidepressants were hydrazine derivatives. However, most of these were withdrawn because, in addition to the cheese reaction, there were serious hepatotoxicity problems. The hepatotoxicity appears to have resulted from the *N*-hydroxylation of the hydrazine group to form a toxic metabolite by microsomal hydroxylases [220]. The one hydrazine, phenelzine (Nardil), that remains in use has few hepatotoxic problems. It appears that the earlier problems may have been compounded by the co-administration of barbiturates to counter sleep disturbances [221]. Such compounds are powerful inducers of the microsomal system and would thus potentiate the toxification of the hydrazine derivatives. The discontinuation of use of barbiturates has now reduced this problem.

Metabolic complications also occur with some other MAO inhibitors. For example, *l*-deprenyl can be metabolized to *l*-amphetamine and it has been speculated that this may contribute to its effects [222]. The formation of the more active enantiomer, d-amphetamine from *d*-deprenyl caused undeniable side-effects that led to led to the discontinuation of that racaemic deprenyl. There is conflicting evidence that the antidepressant tranylcypromine may also be metabolized to amphetamine [223].

In contrast, the antiparkinsonian drugs, *l*-deprenyl and rasagiline are mechanism-based MAO inhibitors with high selectivity for MAO B (>10,000). Effective inhibition of MAO B in the brain by *l*-deprenyl was determined using PET scans as about 60% [37]. l-Deprenyl and rasagiline contain a propargyl group that after oxidation reacts with the flavin prosthetic group forming a covalent adduct at the N5 of the FAD in the active site [18]. In the case of the propargylamine derivatives, their off-target neuroprotective effects, which are independent of MAO inhibition [see 24], enhance their therapeutic value.

### 7.1. Inhibiting Multiple Enzyme Targets

The above description defines an effective approach for therapeutic inhibition of MAO B as irreversible inhibition by a propargylamine derivative to take advantage of the neuroprotective action. In the case of AChE, those known to be effective are reversible, or pseudo-reversible, inhibitors. The use of a single compound to inhibit both MAO B and AChE raises questions about the suitable dosage regime to achieve the correct concentration at the target active sites. In theory, for chronic administration, the reversible inhibition of AChE and BChE can be titrated to the required dose, while MAO inactivation will progress with time. Provided that the selectivity for MAO B is high, then constant low doses of the MAO inactivator should avoid side effects, as has been established in the therapeutic uses of *l*-deprenyl and rasagiline.

Combining two established inhibitors, with known absorption, elimination and distribution kinetics, into a single MTDL can simplify investigations into their behavior in vivo, although it is important to ensure that the MTDL does behave in the same way as its components. This should also take into account that one of the targets, AChE is an extracellular enzyme, whereas MAO is intracellular.

A recent example MTDL is ladostigil, a MAO B–selective propargylamine inhibitor and reversible cholinesterase inhibitor spanning the active and peripheral sites of AChE and BChE, with additional neuroprotective properties [8,224,225]. Although promising in animal studies, the Phase 2 clinical trial did not show significant slowing of the progression of AD. Although ladostigil can give about 80% inhibition of AChE, the major metabolite, R-MCPAI, is a pseudo-reversible inhibitor of AChE due to fast hydrolysis of the adduct, so that inhibition in vivo does not exceed 55% [226]. From the considerations discussed above, this might make it a safer drug candidate.

While exploring simple components for construction of MTDL, one group found that the small propargylamine, *N*-(furan-2-ylmethyl)-*N*-methylprop-2-yn-1-amine (F2MPA), gave enhanced synaptic transmission in the hippocampus of rats. Although it is a very weak competitive inhibitor of human MAO A (IC_50_ = 214 μM) and human MAO B ((IC_50_ = 111 μM), F2MPA increased 5-HT and noradrenaline levels in rat cortex, but with a pattern distinct from those with clorgyline (MAO A) and l-deprenyl (MAO B) [227]. Irreversible inactivation of rat MAO B did occur, with a *K*_I_ value of 65 μM and *k*_inact_ of 0.53 min^−1^. Although binding is much weaker than for *l*-deprenyl, F2MPA is similarly rapid in forming the covalent adduct with FAD. However, metabolism by hydroxylation was fast in the rat, so it seems likely that the increase in rat brain amines was not due to MAO B inhibition. This weak inhibitor of MAO B would be discarded in an in vitro screen, yet the off-target activity of it, or of its metabolites, gave interesting in vivo effects.

Similarly, inhibitors of MAO may also have effects on other monoamine binding proteins, such as the dopamine receptors. The study of promiscuous ligands for the monoaminergic system using off-target predictions for multi-target bioactivities to assess potential MTDL compounds for the treatment of neurological diseases has been reviewed recently [4].

### 7.2. Adding Value to MTDL

Additional aims in compounds to combat neurodegeneration have been to reduce oxidative stress. Inhibiting MAO helps by preventing the formation of H_2_O_2_ and aldehydes, but the main drive has been to incorporate antioxidants or metal chelation activity into the compounds. Recent examples of this strategy have been reported [228,229,230,231,232]. Antioxidants may also help cell survival, so they may be valuable for neuroprotection. For example the MTDLs, MT-031 and MT-031, which combine ChE and MAO inhibitory components, upregulated mRNA levels of the antiapoptotic factor, Bcl-2, the neurotrophic factors, (brain-derived neurotrophic factor (BDNF) and nerve growth factor (NGF), as well as mRNA for the antioxidant enzyme catalase and anti-inflammatory cytokines. In line with the mRNA results, MT-031 was shown to reduce reactive oxygen species accumulation, increase the levels of anti-inflammatory cytokines, IL-10 and decrease the levels of the pro-inflammatory cytokines [230]. Oxidative stress can also arise from poorly functioning mitochondria, whether from ageing or inhibition of the respiratory chain. Neuronal mitochondria maintain function by fusion and fission with defective mitochondria specifically targetted for recycling by mitophagy. Strategies relating to mitochondrial function have been reviewed recently [233,234,235].

## 8. Conclusions

Designing multiple activities into one molecule is a challenge but it may simplify pharmacokinetic evaluation as well as patient drug regimes. However, as the size and complexity of the molecule increases, it gets harder to maintain efficacy at each target. Maintaining specific and potent inhibition of the enzyme and receptor targets is a priority. The physical effects (such as iron chelation of antioxidant properties) will often require higher concentrations in the cell than those required for a selective ligand directed towards to a protein target. This raises the question of combining target actions in the right proportion and the most effective MTDL treatment schedules. For example, the neuroprotective effects of *l*-deprenyl are seen at significantly lower doses lower than those required to inhibit MAO B. In this context, it should be noted that low doses of *l*-deprenyl protect cells from radiation damage, by a process that involves elevation of Bcl-2 levels, but this action is lost at higher doses [236]. Thus, the construction of MTDLs that will prove effective at a single dosage regime may require careful adjustment of the activities of some individual moieties if they are to be a viable alternative to the use of combinations of single drugs. The ability to manipulate specifically the integrated network of signalling pathways that control cell survival is at present not possible, but as targets linked to individual diseases (such as PINK/Parkin to Parkinson’s disease become better understood, the next generation of new target-directed MTDL may be developed.

## Figures and Tables

**Table 1:** Velocity and Michaelis constants for substrates oxidized by purified MAO A and MAO B.

	MAO A			MAO B		
Substrate	*k*_cat_ (s^−1^)	*K*_m_ (mM)	*k*_cat_/*K_m_*	*k*_cat_ (s^−1^)	*K*_m_ (mM)	*k*_cat_/*K*_m_
Benzylamine ^a^	0.02	0.90	0.02	10	0.36	27.8
Phenylethylamine ^a^	0.75	0.50	1.50	3.6	0.067	53.7
Serotonin (5HT) ^a^	2.80	0.40	7.00	0.077	0.28	0.28
Dopamine ^b^	1.83	0.23	7.96			
MPTP ^a^	0.2	0.09	2.22	0.16	0.04	4
Oxygen ^c^		0.06			0.28	
Tyramine ^d^		0.45			0.22	

^a^ Benzylamine, phenylethylamine, serotonin and MPTP from [19], ^b^ Dopamine from [25], ^c^ Oxygen—from [19], ^d^ Tyramine *K*_m_—from [28].

**Table 2:** Some cholinesterase substrates.

Substrate	Enzyme	Application *
Acetylcholine	AChE > BChE	
Acetylthiocholine	AChE > BChE	Substrate used in DTNB assay
Acetyl-β-methyl-choline	AChE	
Acetyl-β-methyl-thiocholine	AChE	Substrate used in DTNB assay
Acetyl-β-methyl-choline	AChE	
Succinylcholine	BChE	
Succinylthiocholine	BChE	Substrate used DTNB assay
Adipoylcholine	BChE	
Benzoylcholine	BChE	
Butyrylcholine	BuChE > AChE	
Butyrylthiocholine	BChE > AChE	Substrate used DTNB assay
Propionylcholine	AChE & BChE	Substrate used DTNB assay
Propionylthiocholine	AChE & BChE	
Phenyl valerate	NTE ^†^ > BChE	Substrate used in NTE assay

^†^ NTE = neuropathy-target esterase. * DTNB 5,5’-dithiobis-2-nitrobenzoic acid (see Section 5).

**Table 3:** Types of enzyme inhibition.

Type	Reaction	Kinetic Constant(s)	Rate of Inhibition	Reversibility In Vitro	Reversibility In Vivo
Reversible	$E+I\;\;\overset{k_{+1}}{\underset{k_{-1}}{⇄}}\;E.I$	*K*_i_ = *k*_−1_/*k*_+1_	Rapid	Dialysis, dilution	Elimination of free I
Irreversible	$E+I\;\overset{k+1}{\to }\;\;E.I$	*k*	Slow	None	Synthesis of new E
Specific irreversible	$E+I\;\;\overset{k_{+1}}{\underset{k_{-1}}{⇄}}\;E.I\;\overset{k_{+3}}{\to }\;\mathrm{E-I}$	*K*_i_ and *k*_+3_	Slow	None	Synthesis of new E
Poor substrate	$E+S\;\overset{k_{+1}}{\underset{k_{-1}}{⇄}}\;E.S\;\cdots \overset{k_{\mathrm{cat}}}{\to }\;E+P$	*K*_i_ and *k*_cat_	Slow	Slow	Substrate depletion
Tight-binding	$E+I\;\overset{k_{+1}}{\underset{k_{-1}}{⇄}}\;E.I$	k_+1_ and *k*_−1_	Slow	Slow	Elimination of free I

**Table 4:** Kinetic behavior of reversible inhibitors.

Type	Effect on *K*_m_	Effect on *V*_max_	Double-Reciprocal Plots at Different [I]
Competitive	*Increased* to *K*_m_ (1 + [I]/*K*_i_)	NONE	Intersect on 1/v axis
Uncompetitive	*Decreased* to *K*_m_/(1 + [I]/*K’*_i_)	*Decreased* to *K*_m_/(1 + [I]/*K’*_i_)	Lines parallel
Noncompetitive	NONE	*Decreased* to *K*_m_/(1 + [I]/*K*’_i_)	Intersect on −1/[S] axis
Mixed	*Increased* to *K*_m_ (1 + [I]/*K*_i_)	*Decreased* to *K*_m_/(1 + [I]/*K’*_i_)	Intersect above 1/v axis if *K*_i_ > *K’*_i_ Intersect below 1/v axis if *K*_i_ < *K’*_i_

**Table 5:** Comparison of percent inhibition and IC_50_ values.

Inhibitor Type	IC_50_	% Inhibition
Competitive	$\frac{K_{i}}{\left[I\right]}\left(1+\frac{\left[S\right]}{K_{m}}\right)$	$\frac{100}{\frac{K_{i}}{\left[I\right]}\left(1+\frac{\left[S\right]}{K_{m}}\right)}$
Uncompetitive	$K_{i}\left(1+\frac{K_{m}}{\left[S\right]}\right)$	$\frac{100}{\frac{K_{i}}{\left[I\right]}\left(1+\frac{K_{m}}{\left[S\right]}\right)}$
Noncompetitive	*K*_i_	$\frac{100}{\left(1+\frac{K_{i}}{\left[I\right]}\right)}$
Mixed	$\frac{\left[S\right]+K_{m}}{\frac{\left[S\right]}{{K^{\prime }}_{i}}+\frac{K_{m}}{K_{i}}}$	$\frac{100}{\frac{\left(K_{m}+\left[S\right]\right)+\frac{K_{m}}{K_{i}}+\frac{\left[S\right]}{{K^{\prime }}_{i}}}{\frac{K_{m}}{K_{i}}+\frac{\left[S\right]}{{K^{\prime }}_{i}}}}$

**Table 6:** Commonly used assay types for MAO. Details are given in the text or in reference [135].

Assay Type	Substrate	Coupling System	Detected Component	Detection
Direct - continuous	Any amine	-	Oxygen	Polarographic (oxygen electrode)
	Benzylamine	-	Benzaldehyde	Absorbance (250 nm)
	Kynuramine	-	4-Hydroxyquinoline	Absorbance (314 nm)
Stopped	Radiolabelled amine	-	Radiolabelled aldehyde	Scintillation counter— after separation
	Most Amines	Aldehyde - derivatization	Aldehyde derivative	HPLC
	Most Amines	Peroxidase	H_2_O_2_ → Dye (e.g., Luminol)	Luminescence
Coupled	Most Amines	Aldehyde dehydrogenase	Aldehyde → NADH_2_	Absorbance (340 nm)
	Most Amines	Peroxidase	H_2_O_2_ → Dye (e.g., Amplex Red)	Fluorescence (e.g., resorufin; λ_ex_ = 535 nm, λ_em_ = 595 nm)

**Table 7:** IC_50_ values for ASS234 (30 min preincubation).

Species	MAO A (nM)	MAO B (nM)	AChE (μM)	BChE (μM)
Eel/Horse ^1^			0.35 ± 0.011	0.46 ± 0.06
Rat ^2^	5.2 ± 1.1	43 ± 8.0		
Human ^3^	0.17 ± 0.03	15830 ± 1040	0.81 ± 0.06	1.82 ± 0.14

^1^ AChE is from electric eel, BChE from horse [183]. ^2^ From [10]. ^3^ From [184].

## Abbreviations

The following abbreviations are used in this manuscript:

ACh	acetylcholine
AChE	acetylcholinesterase
ASDIN	active-site-directed inhibitor
BChE	butyrylcholinesterase
AChEIs	acetylcholinesterase inhibitor
AD	Alzheimer’s disease
ChE	cholinesterase
COMT	catechol-*O*-methyltransferase
DA	dopamine
DFP	diisopropylfluoro phosphate (diisopropylphosphofluoridate)
L-DOPA	L-3,4-dihydroxyphenylalanine (dopamine precursor)
DTNB	5,5’-dithio-bis-[2-nitrobenzoic acid]
FAD	flavin adenine dinucleotide
5-HT	5-hydroxytryptamine (serotonin)
MAO	monoamine oxidase
MAOI	monoamine oxidase inhibitor
MTDL	multitarget-directed ligand
NTE	neuropathy-target esterase
OP	organophosphate
PAS	peripheral anionic site
PD	Parkinson’s disease
PS	presenilin
QSAR	quantitative structure-activity relationship
TNB	2-nitro-5-thiobenzoate
